# Investigation on the effects of the application of a sublimating matte coating in optical coordinate measurement of additively manufactured parts

**DOI:** 10.1007/s00170-025-16378-x

**Published:** 2025-09-03

**Authors:** Sofia Catalucci, Tomáš Koutecký, Nicola Senin, Samanta Piano

**Affiliations:** 1 Department of Industrial Engineering, University of Padua, Padua, Italy; 2 Manufacturing Metrology Team, Faculty of Engineering, University of Nottingham, Nottingham, UK; 3 Institute of Machine and Industrial Design, Faculty of Mechanical Engineering, Brno University of Technology, Brno, Czech Republic; 4 Department of Engineering, University of Perugia, Perugia, Italy

**Keywords:** Matte coating, Sublimating coating, Coating thickness, Performance indicators, Measurement error

## Abstract

Coating sprays play a crucial role in extending the capabilities of optical measuring systems, especially when dealing with reflective surfaces, where excessive reflections, caused by incident light hitting the object surface, lead to increased noise and missing data points in the measurement results. This work focuses on metal additively manufactured parts, and explores how the application of a sublimating matting spray on the measured surfaces can improve measurement performance. The use of sublimating matting sprays is a recent development for achieving temporary coatings that are useful for measurement, but then disappear in the final product. A series of experiments was performed involving measurement by fringe projection on a selected test part pre- and post-application of a sublimating coating layer. A comparison of measurement performance across the experiments was run by computing a selected set of custom-developed point cloud quality indicators: rate of surface coverage, level of sampling density, local point dispersion, variation of selected linear dimensions computed from the point clouds. In addition, measurements were performed using an optical profilometer on the coated and uncoated surfaces to determine both thickness of the coating layer and changes of surface texture (matte effect) due to the presence of the coating layer.

## Introduction

Additive manufacturing (AM) is a production technology for the fabrication of complex freeform parts adopted nowadays by many manufacturing industries in the automotive, aerospace, biomedical, electronic and construction sectors. The increased application of AM in the fabrication of industrially relevant parts is raising the bar for quality inspection, leading to stricter measurement performance requirements [1–3]. In tandem with traceable contact methods, optical coordinate measuring technologies have been massively used for the inspection of AM parts, given the advantages of increased sampling density, speed and flexibility of operation. However, when measuring metal AM parts, optical methods struggle when encountering highly reflective surface features (common with steel and titanium alloy parts) and may frequently fail to acquire points. As an example, when using systems based on fringe projection, the resulting reflections generated by the light hitting the surface of the measured objects lead to pixel saturation and incorrect decoding, causing noise and missing data [4]. One approach to minimising reflections in 3D scanning involves high dynamic range (HDR) techniques, where multiple images with different exposure times are combined to retain only the highest unsaturated pixel values [5–7]. Recent work by Liu et al. showed that an HDR-like pseudo-exposure fusion can restore up to 45% of missing fringes on mirror-finished steel parts [8], while Feng et al. extended the same concept to multi-view setups with CNN-based quality metrics [9]. Alternatively, structured light methods using binary patterns, as proposed by Gupta et al. [10], mitigate indirect illumination effects by reducing subsurface scattering, inter-reflections, and diffusion, improving measurement performance on reflective surfaces. In parallel, complementary approaches such as anti-glare paints and low-damage cryogenic polishing have been explored [11, 12] in order to mitigate the effects of surface reflectivity and as-built roughness that would affect the accuracy and completeness of optical scans.

The methods mentioned so far are often only able to partially minimise the reflectivity issue. For this reason, a widely used alternative is the employment of special matting sprays. For sufficiently small parts (i.e., tens of millimetres) a suspension of titanium dioxide (TiO_2_) with ethanol is often used, while for larger parts (i.e., millimetres to metres) chalk sprays are usually employed. For what concerns the measurement of highly challenging materials, Semmes et al. [13] have addressed the issue of acquiring transparent microdevices using an optical profilometer. In their study, they used a low-cost TiO_2_ nanoparticle suspension to opacify with a handheld airbrush the surface of a 3D printed polymeric object. They performed a comparison in terms of thickness, time efficiency, and cost efficiency of various concentrations of the custom TiO_2_ spray, as well as commercially available 3D scanning sprays. Research on matte coatings has explored their impact on 3D scanning accuracy, particularly fringe projection and laser-based sensors, with studies examining different materials, coating thicknesses, and application methods. Owczarek et al. [14] recently provided a preliminary metrological study on the effects on optical measurements of different matting strategies and coating sprays of different properties (sublimating and non-sublimating). Several reference spheres were measured to check the influence of the material on the results with and without coating. The results showed that ultra-thin coating layers can cut coverage loss by approx. 30%. Advances such as ultrasonic atomisation have enabled submicron coatings, improving homogeneity and reducing reflectivity without excessive material build-up. Paloušek et al. [15] studied the thickness of the matting spray created by hand-applied TiO_2_ and chalk powder coating onto glossy surfaces. Koutecký et al. [16], Hruboš et al. [17] and Franke et al. [18] investigated the effects of TiO_2_ spray and how the matting layer affects the quality of the measurements; the coating was applied using an automatic coating system and a specifically designed atomiser spray gun based on the work of Rukosuyev et al. [19]. However, challenges remain in balancing reflectivity reduction with ease of coating removal, as in the case of TiO₂ coatings; although effective, this kind of coatings are difficult to clean from surfaces, as it was demonstrated in [20].

As found in the literature, the main disadvantages of using coating materials such as TiO_2_ are the potential health risks for the operator to inhale the substances [21] and the difficulty in removing the material itself from the surface of the part, especially when the part is not smooth [20]. For these reasons, sublimation matting sprays have made their appearance in the past few years and have been increasingly used in 3D optical measurements of additively manufactured parts [22]. These sprays are based on cyclododecane or related substances, and they fully sublimate from the surface of the part after a certain period. In the available literature, sublimation matting sprays have only marginally been subjected to research. Díaz-Marín et al. [23] used cyclododecane spray for the digitisation of archaeological glass artefacts measured using optical instruments. In this specific case, other coating materials could not be used, given the risk of potentially damaging the archaeological parts when removing the spray from the surface. The authors investigated the differences in the results between opacified (when using the cyclododecane spray) and clear glass archaeological artefacts based on the obtained number of measured points. In addition, they evaluated the measurement error caused by the use of the spray, comparing cyclododecane and conventional matting materials applied to the surfaces of a calibrated artefact and two objects with complex shapes. The results showed the suitability of cyclododecane in aiding the optical instrument to optimally acquire the selected artefacts. Franke et al. [24, 25] compared the layer thicknesses and sublimation times of sublimation matting sprays available from different manufacturers. The authors carried out the investigation on perfectly planar and smooth objects (such as Si-wafer and gauge block) using a 3D structured light scanner and an industrial camera (for the sublimation times). The results showed the specific values of the deposited layer thicknesses and the actual sublimation times for each material.

### Contents and research motivation

Optical systems struggle when they face the shiny, rough surfaces that are typical of metal parts made by AM. Standard anti-glare sprays based on permanent powders solve the reflection problem but bring two drawbacks: (a) the powder can be harmful if inhaled and (b) it clings to every recess, so removing it from a complex part is slow, costly, and may leave residuals. On the other hand, sublimating matte sprays form a thin crystalline layer of only a few micrometres thickness that disappears within a few to tens of hours. The temporary nature of these coatings eliminates operator exposure during removal and prevents contamination. Despite their widespread use in industry, their performance has been quantified only on smooth, planar specimens.

The aim of this work is to verify if the use of sublimation matting spray can help improve the performance of optical coordinate measurement of metal AM parts. Metal AM surfaces feature irregular, complex topographies that are already naturally challenging to acquire by optical means. Such surfaces become even more challenging to measure when the materials feature high reflectance, as it is often the case with metal AM. Therefore, this work focused on estimating surface measurement performance achievable by optical measurement of metal AM parts with and without a specific type of sublimating matte coating. Performance was quantified by evaluating and comparing several custom indicators developed in previous works [26–28]. These indicators specifically capture: (i) the capability of measurement to adequately cover all the surfaces of the part with a sufficient number of points; (ii) the local spatial density of point-based measurement; and (iii) the local point dispersion measured along the local surface normal, as a measure of precision across measurement repeats. Details of the coating application strategy, the indicators applied in the measurement campaigns and the measurement setup are reported in Sect. 2. An additional goal of this work was the determination of how surface topography changes due to the added coating layer, as reported in Sect. 2.5. An overarching challenge in this work was that the coating begins sublimating immediately after deposition, which led to designing specific experiments aimed at determining how long does it take for the coating to disappear and for the surface to lose its desired optical properties. The rest of the paper is structured as follows: Sect. 3 presents the experimental results of the applied indicators (3.1 to 3.3), comparing the three coating strategies across both laboratories and setups. Sections 3.4 and 3.5 analyse the surface texture data. Section 4 discusses and synthesises the findings. Finally, Sect. 5 concludes the study and highlights advantages, limitations and avenues for future work.

## Materials and methods

The measurement campaigns were performed using two fringe projection instruments of different performance (for instance, 5 MP versus 8 MP cameras), one available at the University of Nottingham (UoN) and another one at Brno University of Technology (BUT), respectively. Measurements were taken pre and post application of a coating layer onto the surface of a metal AM part with a spray gun. More specifically, the following three measurement and spray application strategies were adopted, as reported in Table 1 below.

**Table 1:** Spray application strategies

Strategy	Coating routine	N. of repeats	Purpose	Notation
A	No coating application	4	Baseline	UoN 0 or BUT 0
B	Single coating application, execution of four measurement repeats	4	Tracks performance decay as the layer sublimates	UoN 1 or BUT 1
C	Coating application, execution of one measurement; the same sequence of two actions repeated four times	4	Measures the best performance achievable with a fresh coating	UoN 4 or BUT 4

### Performance indicators

**Fig. 1:**
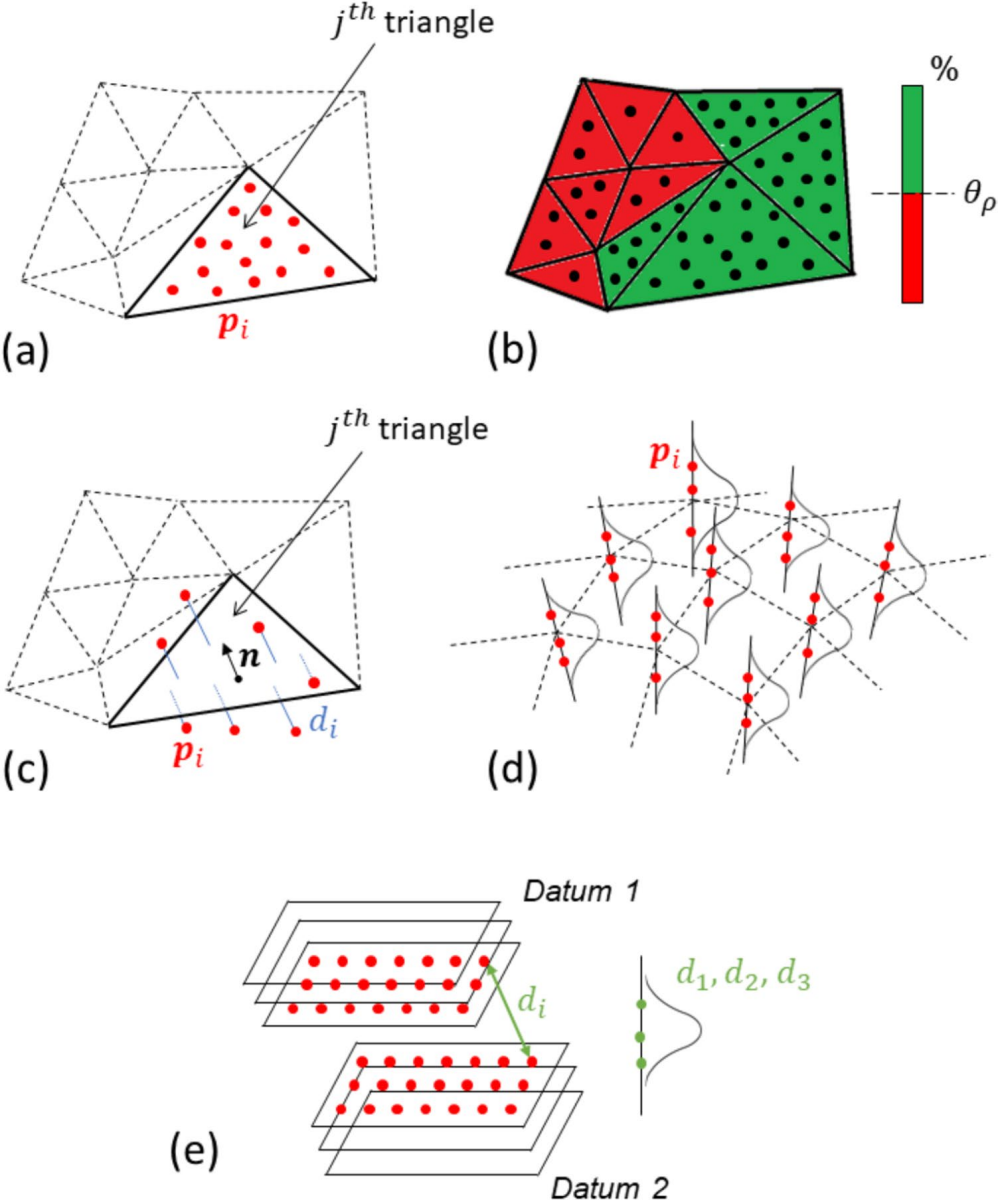
Schematic representation of the performance indicators: (**a**) local sampling density (${\rho }_{j}$), (**b**) part coverage (${c}_{A}$ and ${c}_{n}$), **(c)** local sample dispersion (${s}_{j}$), (**d**) local dispersion in the Gaussian field (${{\sigma }^{2}}_{GF}$), and (**e**) dispersion in linear dimensions

The following set of custom-designed performance indicators developed in previous work [26, 27], inspired by the work carried out by [29–32], was used to quantify measurement performance. A comprehensive review of performance indicators for point cloud quality can be found elsewhere [33]. All the indicators (shown in Fig. 1) require that the measured point clouds are first registered with respect to a reference geometric model representing the measurand, typically provided in the form of a triangle mesh, exported directly from the available CAD model. The mesh step size for tessellation was fixed only for computational practicality, and the study of the influence of mesh parameters on the indicator values is out of the scope of this work. The same mesh was used for all coating strategies and in both campaigns, ensuring that any discretisation bias is eliminated, making the spray application’s effect the sole variable under investigation.

The description of the proposed indicators is as follows:

– Local sampling density is the number of measured points per surface area [26]. It is a “local” indicator in that it refers to a region of the measured surface. If the reference is a triangle mesh, the local sampling density is computed for each triangle. For the ${j}^{th}$ triangle, belonging to a mesh of ${n}_{\mathrm{t}\mathrm{r}\mathrm{i}}$ triangles, the local sampling density ${\rho }_{j}$ is the number of points ${n}_{\mathrm{p}\mathrm{t}\mathrm{s},\mathrm{j}}$ acquired from the surface region occupied by the triangle, divided by the area of the triangle itself of area ${A}_{j}$ (Fig. 1(a)):

1 $${\rho }_{j}=\frac{{n}_{\mathrm{pts},\mathrm{j}}}{{A}_{j}}$$

The local sampling density indicator is designed to be computed over the point cloud resulting from a single measurement process. The same indicator can also be computed on measurement repeats to assess how stable the sampling density is in repeatability/reproducibility conditions.

– Part coverage expresses the capability of a measurement to adequately cover all the surfaces of the part with a sufficient number of points. This is a “global” indicator (as opposed to local) and is quantified as the ratio of surface area covered with sufficient sampling density (defined above) over the total surface area [26]. Note that “sufficiency” of local sampling density is defined by setting a threshold on local spatial density values (i.e., areas whose sampling density is greater or equal to a given threshold value are classified as covered, otherwise are classified as uncovered). If the reference geometry is provided as a triangle mesh of ${n}_{\mathrm{t}\mathrm{r}\mathrm{i}}$ triangles and ${\theta }_{\rho }$ is the pre-set global threshold on the local sampling density, then the ${j}^{th}$ triangle is sufficiently covered if its local sampling density is equal or above the threshold. In this work ${\theta }_{\rho }$ was set to 75% of the maximum detected local sampling density across each point cloud, calculated as the point count at each facet divided by the facet surface area. In practice, an adequate threshold value should be chosen by the user depending on application and measurement requirements. Part coverage can be expressed as the ratio ${c}_{A}$: total area of sufficiently covered triangles over total area of all triangles, reported as a percentage:

2 $${c}_{A}= \frac{\sum_{jj=1}^{{n}_{ctri}}{A}_{jj}}{{\sum }_{j=1}^{{n}_{\mathrm{tri}}}{A}_{j}}\bullet 100$$

where $j$ is an index covering all the triangles in the mesh, $jj$ is a subset of $j$ indicating sufficiently covered triangles (i.e.: ${\rho }_{jj}\ge {\theta }_{\rho }$) and ${n}_{ctri}$ is the total number of sufficiently covered triangles. Alternatively, part coverage can be indicated as a ratio of triangle counts ${c}_{n}$, defined as the number of sufficiently covered triangles over total number of triangles, also reported as a percentage:

3 $${c}_{n}= \frac{{n}_{\mathrm{ctri}}}{{n}_{\mathrm{tri}}}\bullet 100$$

The two indicators of part coverage are visually summarised in Fig. 1(b). Note that the ${c}_{n}$ indicator provides a reliable indication of coverage only for uniform meshes, where the areas of the triangles are all very similar to each other. This indicator is defined purely as a triangle-count ratio (i.e., the percentage of facets that meet the minimum sampling density threshold) so it does not directly depend on triangle size.

As for the local sampling density indicator, the part coverage indicators are designed to be computed over a single measurement repeat. Observing how these indicators vary across repeats can be used to assess part coverage variation in repeatability/reproducibility conditions.

- Local dispersion: the term “dispersion” refers to positional scatter of measured points, where the scatter is measured orthogonally to a reference surface (i.e., “vertical” scatter as opposed to “lateral” scatter, in a local reference frame). The indicator is local as it refers to a region of the measured surface. For triangle meshes, scatter is local to each triangle and is measured along the triangle normal [26, 27]. Local point dispersion can be expressed through two indicators, differentiated by how the reference surface and the actual scatter are computed: where ${n}_{pts,j}$ is the number of points that are associated to the ${j}^{th}$ triangle. The expression also indicates that ${s}_{j}$ is the sample standard deviation of the “vertical” positions of all the measured points associated to the ${j}^{th}$ triangle (assuming the triangle surface as the 0 coordinate of the axis normal to the triangle). Note that ${s}_{j}$ is the dispersion of a sample (the measured points associated to a triangle in the mesh) and is neither representative of population parameters, nor it captures spatial dependencies amongst measured points.
- Local sample dispersion: measured orthogonally to the surface of each triangle. With ${d}_{i}$ being the signed Euclidean distance of each measured point ${{\boldsymbol{p}}}_{i}$ to the surface of the associated ${j}^{th}$ triangle, local sample dispersion ${s}_{j}$ is computed as the mean distance of all the points associated to that triangle [26] (Fig. 1(c)): where ${n}_{pts,j}$ is the number of points that are associated to the ${j}^{th}$ triangle. The expression also indicates that ${s}_{j}$ is the sample standard deviation of the “vertical” positions of all the measured points associated to the ${j}^{th}$ triangle (assuming the triangle surface as the 0 coordinate of the axis normal to the triangle). Note that ${s}_{j}$ is the dispersion of a sample (the measured points associated to a triangle in the mesh) and is neither representative of population parameters, nor it captures spatial dependencies amongst measured points.
  4 $${s}_{j}=\frac{\sum_{i=1}^{{n}_{pts,j}}{d}_{i}}{{n}_{pts,j}}$$
- Local dispersion in the Gaussian field: in this rather more involved indicator, the measured points are fitted to a Gaussian field so that spatial dependencies between neighbouring points can be captured [27, 28]. For a fully three-dimensional triangle mesh, the field is wrapped around the measured geometry so that the local “vertical” direction of the field is always locally orthogonal to the mesh. Once the field has been fitted to the measured points [27, 28], it can be interrogated at any position in space to extract the local variance ${{\sigma }^{2}}_{GF}$, which is a measure of local dispersion (Fig. 1(d)). Similar to ${s}_{j}$, ${{\sigma }^{2}}_{GF}$ expresses local dispersion of measured points, computed orthogonally to the surface; however, the latter incorporates spatial dependencies between neighbouring points.
  The indicators of local dispersion can be computed over a single measurement repeat or using multiple measurement repeats in repeatability or reproducibility conditions. In the former case, the dispersion indicators will provide an indication of how scattered measurement points are with respect to where they are taken on the measurand surface (i.e., in correspondence to one triangle vs another). In the latter case (measurement repeats), the indicators provide insight on the combined effects of both point localisation (i.e., where points are taken) and random measurement error due to measurement repetition (in repeatability or reproducibility conditions).
- Dispersion in linear dimensions: this is an indicator that can only be computed using measurement repeats. Contrarily to all the previous ones, this indicator is also not associated directly to properties of the measured points or their spatial distributions, but is rather associated with linear dimensions (distances, lengths, diameters) computed via point cloud segmentation and datum fitting, starting from the measured points. For each measurement repeat, the segmentation/datum fitting process is re-run for the feature of interest, using the point clouds of each measurement repeat, and the obtained linear dimension values are accumulated until there is a large enough number of them to compute the dispersion of the value. If measurement repeats are available in low numbers, they can be used to fit a Gaussian field and the latter can be used to generate new artificial repeats by Monte Carlo simulation [27] (Fig. 1(e)). The dispersion of linear dimension values indicator directly provides a measurement of the quality of a dimensional assessment result performed from the measurement, which is one of the typical targets of geometric/dimensional inspection.

### Designed test artefact

**Fig. 2:**
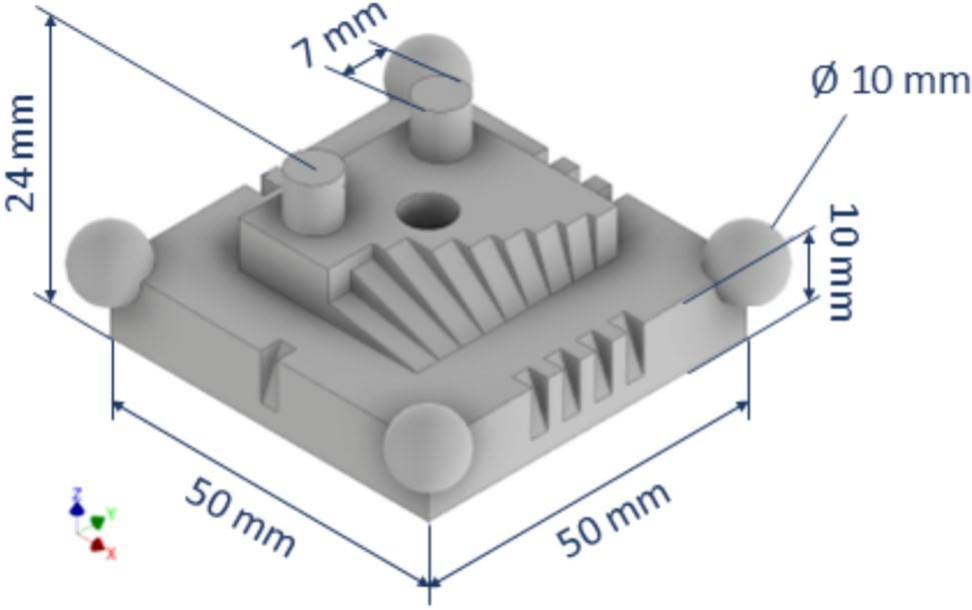
Designed test artefact

All the experiments were conducted on a custom test artefact fabricated by laser powder bed fusion (PBF-LB) and designed to replicate the most common types of surfaces and orientations encountered on parts fabricated by said additive technology (Fig. 2). The test artefact features a square base with one to four notches located at the four sides in order to track angular orientation. Four spheres are located at the base corners. The raised, central region of the artefact features two cylindrical protrusions and a cylindrical slot hole, as well as a series of angled steps with slopes ranging from 10° to 90° (vertical). Several linear dimensions are provided as targets for the performance indicator related to dimensional dispersion (see previous section).

**Fig. 3:**
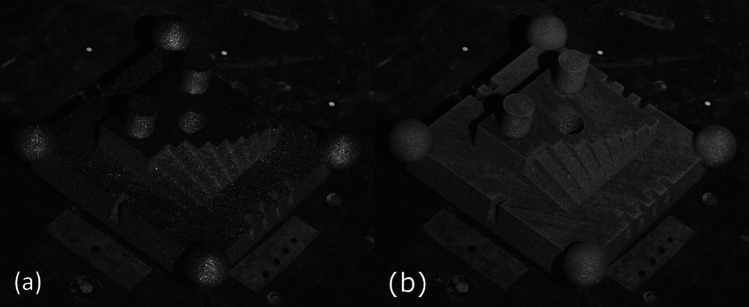
Views of the test artefact from the camera of the optical instrument at UoN pre (**a**) and post (**b**) coating application

The test artefact was produced in 316L stainless steel using a Renishaw AM125 located in the Centre for Additive Manufacturing (CfAM) at UoN. Two images acquired from the fringe projection system used in this work show the view of the test artefact from the cameras of the optical instrument at UoN (pre and post coating application in Fig. 3(a) and (b), respectively).

The test artefact was designed to replicate the principal metrological challenges faced by optical systems during routine inspection of metal AM components. First, its surface exhibits the high reflectivity and light-scattering behaviour that characterise many AM parts. Then, geometrically, the artefact mixes nominally flat regions with steep flanks, deep recesses, and a through-slot, thereby imposing wide variations in incidence angle and frequent self-occlusions, both common causes of data gaps in measurements. Finally, it integrates standard reference features, such as four corner spheres, three internal cylinders, and two orthogonal datum planes, allowing any bias or dispersion introduced by coatings or optics to be quantified on dimensions routinely used for quality control.

### Sublimating matte coating application

**Fig. 4:**
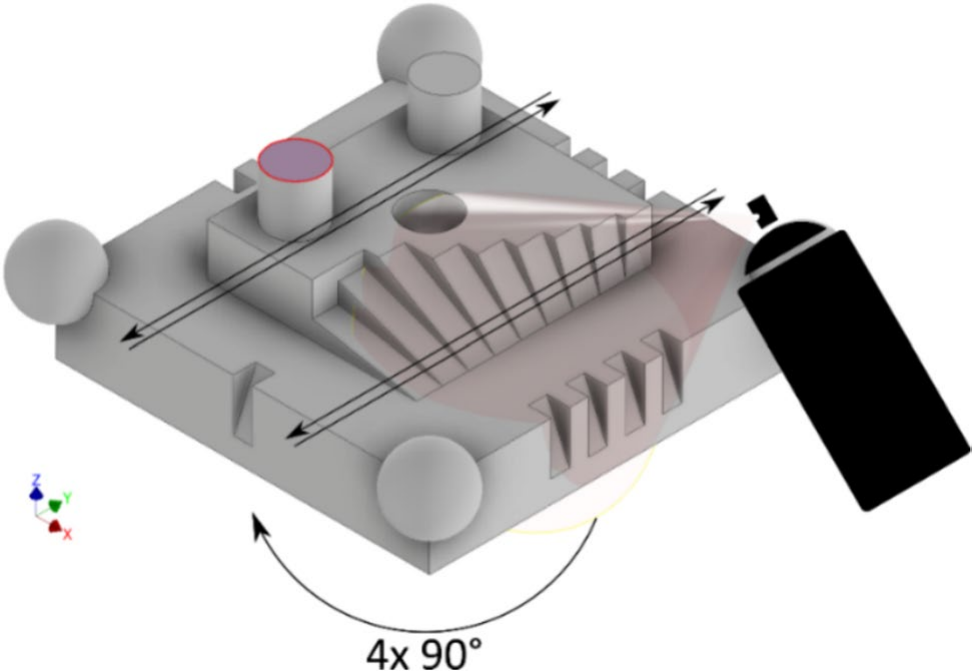
The process of applying the matting spray to the artefact to ensure that all visible surfaces were evenly covered. The top surface of the cylinder (highlighted in red) indicates the area of the artefact analysed to capture a possible change in surface texture characteristics (see later Sect. 2.5)

AESUB Orange scanning spray was used in this work as test matting spray. AESUB Orange is specifically formulated for its sublimating nature, controlled thickness of the coating layer, and no pigment residue is left, unlike traditional powders or sprays. The spray is a self-volatilising material without any pigment based on hydrocarbon compounds. The spray crystallizes during the application process and then sublimates spontaneously over time, eventually leaving no residual material on the surface. The manufacturer’s specified thickness of a sprayed layer is between 2 and 6 µm; the time window to perform optical scans post spraying is between 4 and 8 h, given an estimated sublimation time of 12 to 24 h, as suggested by the spray manufacturer. The spraying material is supplied as canned aerosol and was applied from a constant distance of approx. 150 mm at a 45° angle to the top surface. The artefact was then rotated in 90° increments through four orientations as shown in Fig. 4, and the back-and-forth spray path was repeated twice (once in each rotation direction) to achieve an even matte layer on all visible areas. The Si-wafer was sprayed in a similar manner as the artefact. The distance was approximately 150 mm, the angle was 45°, and 8 spray passes were made over the Si-wafer, which should closely match the number of passes over the top surface of the cylinder.

### Measurement setup

Two optical coordinate measuring instruments were selected to carry out the experimental measurement tests. The first was a commercial fringe projection system GOM ATOS Core 300 available in the Manufacturing Metrology Team laboratory at UoN. The room was temperature-controlled at $\left(20\pm 0.5\right)^\circ{\rm C}$. The field of view was (300 × 230) mm with an average distance between two measured points (i.e., point sampling) of 0.12 mm. The second fringe projection system was a GOM ATOS III Triple Scan, available in the Optical Digitization and Quality Control laboratory at the Brno University of Technology (BUT), temperature-controlled at $\left(21\pm 1\right)^\circ{\rm C}$. The field of view was (170 × 130) mm with point sampling of 0.055 mm. The parameters obtained by the manufacturer Acceptance Test are reported in Table 2, while specifications and scanning settings per instrument are reported in Table 3.

**Table 2:** Parameters obtained by the manufacturer Acceptance Test

	UoN fringe projection system	BUT fringe projection system
Probing error form (sigma)	0.006 mm	0.003 mm
Probing error size	0.027 mm	0.006 mm
Sphere spacing error	0.020 mm	0.008 mm
Length measurement error	0.047 mm	0.014 mm

**Table 3:** Systems specifications and scan point computation settings

	UoN fringe projection system	BUT fringe projection system
Field of view	(300 × 230) mm	(170 × 130) mm
Point sampling	0.12 mm	0.055 mm
Camera resolution	5 MP	8 MP
Min. fringe contrast	10	15
Points at strong brightness differences	Use	Avoid
Points in shadow areas	Use	Use
Points in groove edges	Use	Avoid
Triple Scan points	Avoid	Use
Triple Scan points at strong brightness differences	N/A	Avoid
Max. residual	0.30 pixel	0.20 pixel
Max. viewing angle sensor/surface	80°	80°

Eight scanning positions with angular spacing of 45° between them were planned for both measuring instruments. Both instruments were positioned at a distance so that the centre of the artefact is approximately in the cameras’ focal plane, and tilted at 45° to face as much of the surface of the artefact as possible. For all repeated measurements, the artefact was placed at the same initial position and orientation: at UoN using a manual rotary stage that allows precise positioning, at BUT using an automated rotary stage. Eight reference points (markers), placed evenly around the artefact on the rotary stage both at UoN and at BUT, were used to automatically align the individual scans into a common coordinate system using the commercial software available. For measurements executed post coating application, the time interval between the coating application and the start of the measurement was between 2 and 4 min, and the total scanning time was 4 to 6 min per repeat. Table 4 reports a simplified visual representation of the scanning orientations and sensor’s positioning (both top and lateral views).

**Table 4:**
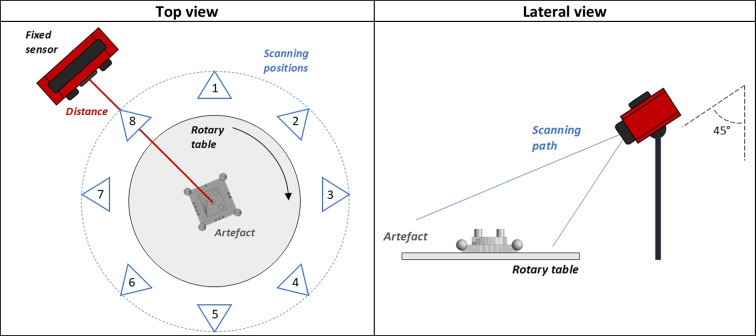
Schematic representation of measuring positions for both campaigns

### Coating thickness and surface texture

Further experiments were conducted to evaluate achievable thicknesses for a given spraying time and to assess how the topography of the surface changed from uncoated to coated state, in particular to appreciate the loss of reflective power (matte effect).

**Fig. 5:**
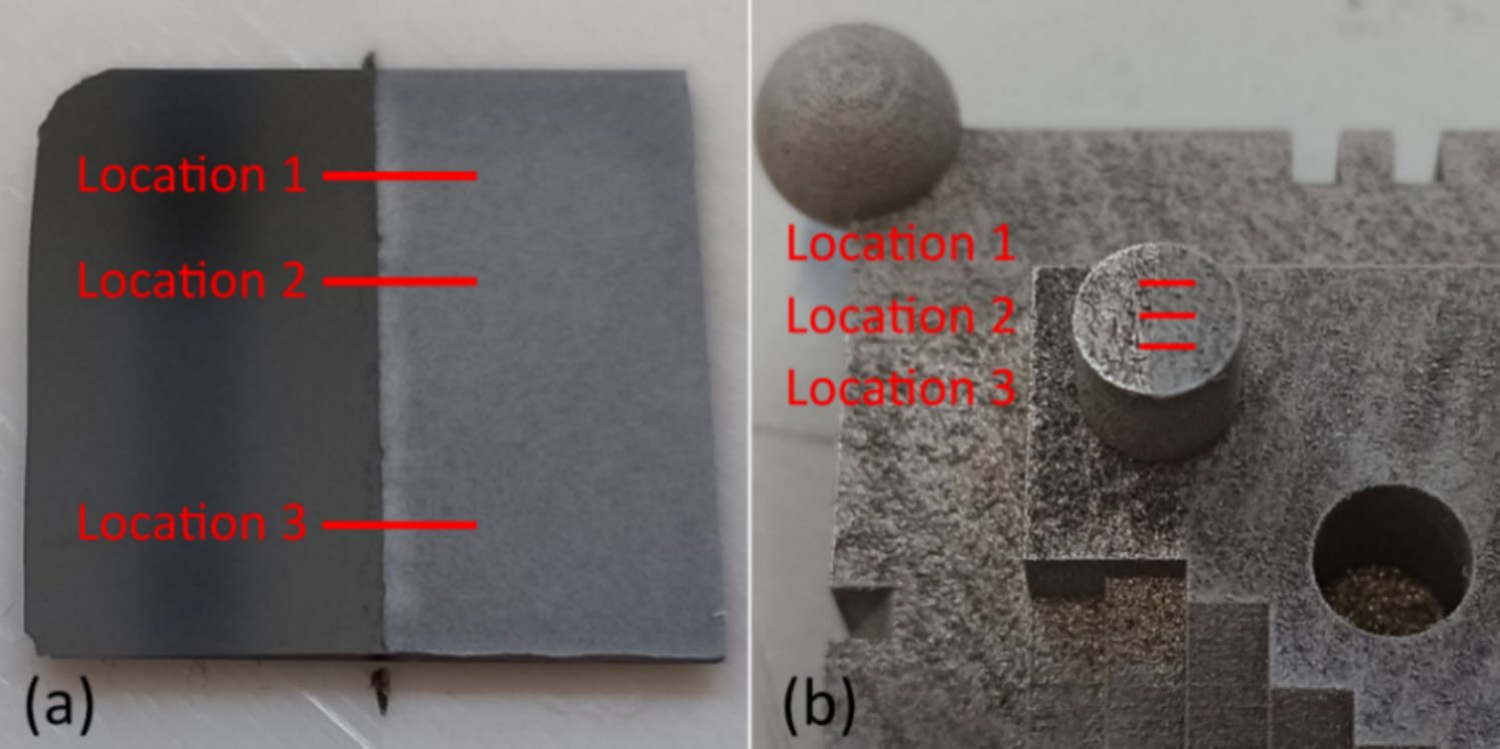
Matte coated surface after removal of the paper tape with indication of three measurement locations. (**a**) Si-wafer. (**b**) Top surface of a cylindrical feature in the test artefact

**Fig. 6:**
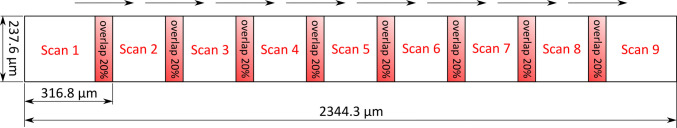
The stitching of nine consecutive topographical measurements of (237.6 × 316.8) µm acquisition area with a 20% overlap, for a total length of 2344.3 µm. The direction is indicated by the arrows

Changes in the surface texture characteristics caused by the coating application were analysed on the top surface of one of the protruding cylinders in the test artefact (Fig. 4). In addition, the flat and smooth surface of a Si-wafer was coated using the same process to act as a comparison reference. For both the top surface of the protruding cylinder and the wafer surface, paper tape was used to cover half of the surface during spraying and removed after application (Fig. 5). Surface texture characteristics and coating thickness were measured using a Bruker Contour GT-X optical profilometer with system parameters and settings indicated in Table 5. The measuring procedure used to obtain the data with the profilometer is represented in Fig. 6.

**Table 5:** Parameters of 3D optical profilometer Bruker Contour GT-X

**Parameters**		
Vertical resolution	0.05 nm	
Lateral resolution	0.495 µm	
**Measurement setup**		
Objective	20 ×	
Magnification	20 ×	
Measurement area	2.3 × 0.24 mm (using stitching with 20% overlap)	
Scanning speed	1 ×	
Threshold	2%	
Illumination	Green light (narrow band)	
**Analysis setup**	**Coating thickness (Si-wafer)**	**Surface texture (artefact)**
Levelling	Tilt only + Zero mean	Zero mean
Gaussian regression filter	-	S-filter: 0.025 mm

For measuring the coating thickness, the captured uncoated part of the Si-wafer surface was used for levelling the data to zero and removing any tilting. Thus, it was possible to measure the resulting coating thickness on the matted area of the Si-wafer in the three locations indicated in Fig. 5a and, equally, in the three locations indicated in Fig. 5b of the selected feature surface on the artefact. Locations were chosen at a sufficient distance from the edges of both samples and at random intervals between them. The time interval between the realisation of the coating application and the beginning of the measurements with the optical profilometer was 2 to 4 min. The measuring process at each location lasted approximately 6 min, leading to a total of 18 min needed to measure both the Si-wafer and the test artefact.

## Results

Despite the difference in the scanning settings reported in Table 3 in Sect. 2.4 and instruments employed, preliminary visual inspection of the results (Table 6) indicated an immediate improvement when using the spray.

**Table 6:**
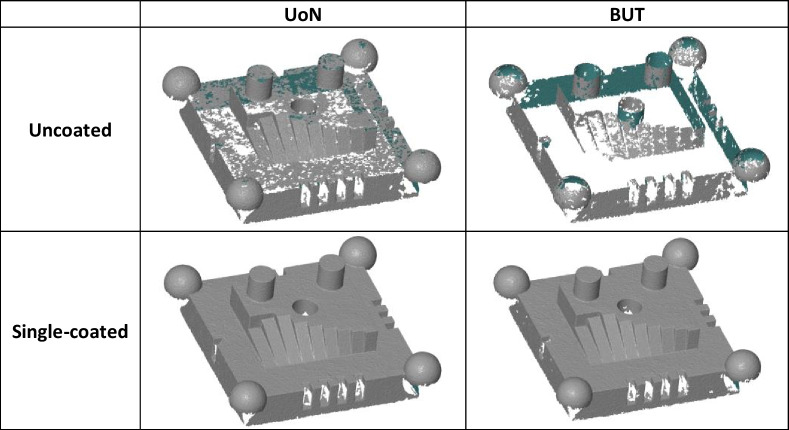
Measured points despite the difference in instrument/scanning settings, pre and post coating

Given the high computational cost of processing point clouds with thousands of points, all original datasets were downsampled from 500,000 to 20,000 points approx. using the grid average method introduced by [34] to achieve 0.5 mm target point spacing. The reference geometric model needed to compute the selected custom-designed performance indicators (see Sect. 2 and previous work [26, 27]) was provided in the form of a triangle mesh in STL format. The measured point clouds were registered to the triangle mesh using a two-step sequence consisting of coarse alignment via feature-matching Fast Point Feature Histograms (FPFH [35]) and fine alignment by Iterative Closest Point (ICP [36]) between a fixed dataset (represented by the vertices extracted from the mesh geometry) set as target, and the point clouds (moving dataset). Registration was implemented as a rigid transformation (i.e., rotation and translation only, no scaling). More specifically, in the first phase, registration is achieved via local identification of point features and subsequent clustering into groups with associated similarity/difference metrics. Iterative matching of the identified correspondences is performed via RANSAC algorithm [37]. The result obtained from the transformation represents an initial coarse guess of the point cloud position with respect to the reference geometry. The second registration phase (fine registration) is done using ICP algorithm, refining the previously obtained coarse transformation via iteratively minimising the distance between the fixed and moving datasets.

### Local sampling density, part coverage and local sample dispersion

Colourmaps and statistical distributions for each indicator are presented in this section. In each figure, strategy A refers to the average of four measurements executed in sequence on the uncoated sample; strategy B refers to the average of four measurements executed in sequence after the application of one coating pass; strategy C refers to the average of four measurements, each executed after the application of a new coating pass (i.e., coating was repeated four times, ahead of each measurement). These strategies are reported in Table 1. In each panel of colourmap figures, both results for UoN and BUT are reported.

**Fig. 7:**
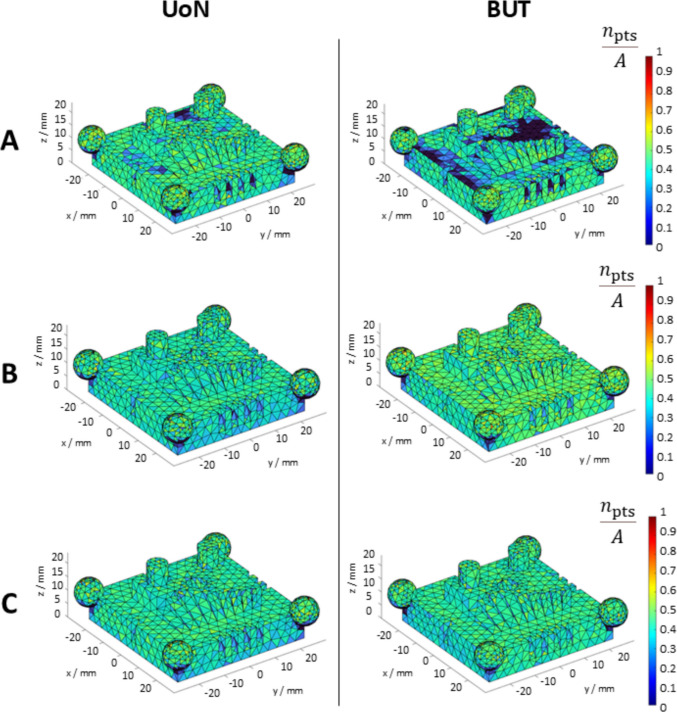
Example maps of triangles coloured on local sampling density ${\rho }_{j}$. All local sampling density values averaged over four repeats for UoN and BUT respectively. Strategies **A**, **B**, **C** refer to Table 1

The example colourmaps of sampling density (i.e., in this work the number of points ${n}_{\mathrm{pts}}$ associated with each triangle in the mesh divided by the total area of the triangle) are shown in Fig. 7. The outcome per triangle was averaged over four sampling density values obtained from the respective four measurement repeats. Values were normalised by division with the maximum sampling density value recorded across all datasets.

**Fig. 8:**
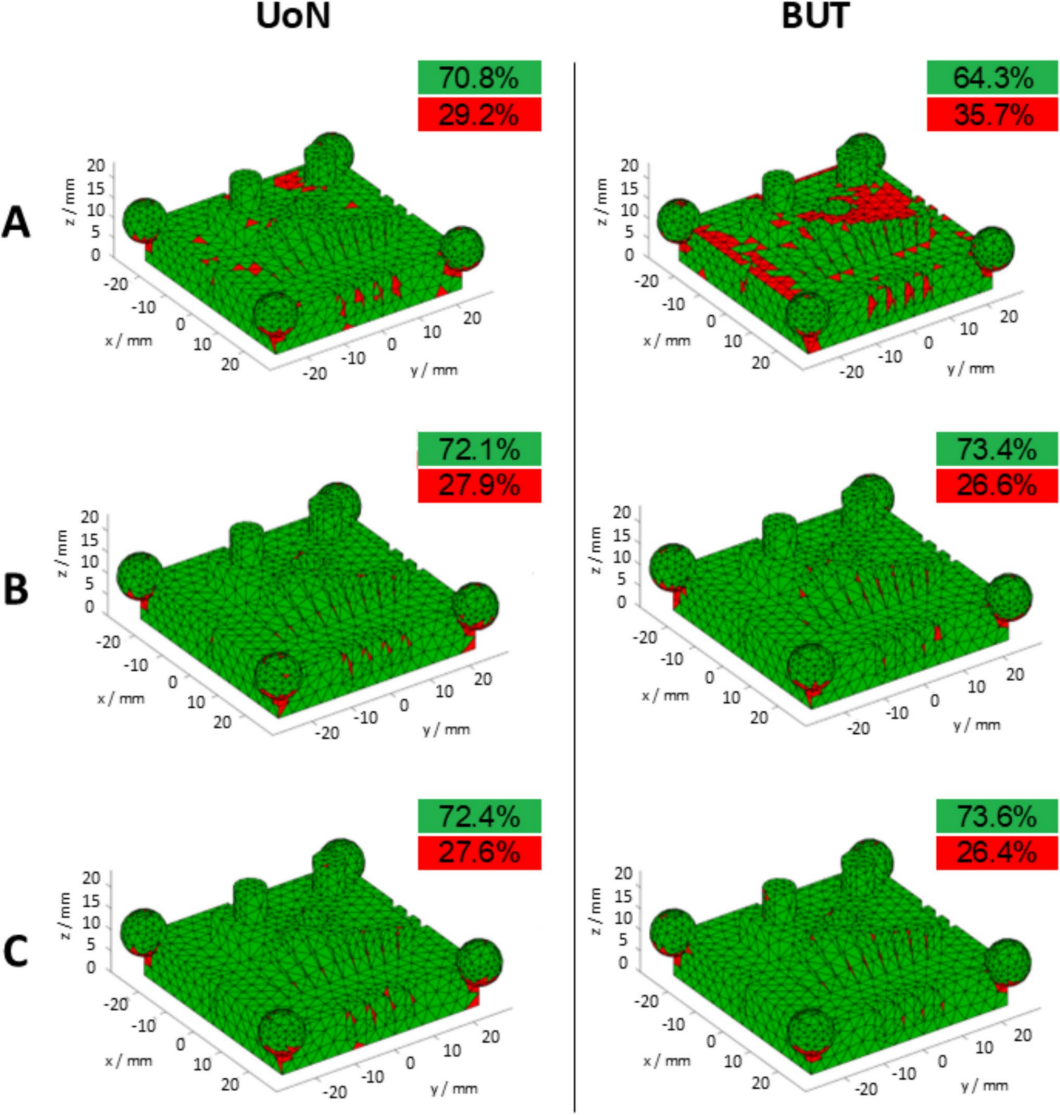
Part coverage ${c}_{n}$: triangle mesh of the measurand artefact, coloured using thresholded local sampling density (green: sufficiently covered triangles; red: insufficiently covered triangles). All local sampling density values averaged over four repeats for UoN and BUT, respectively. Strategies **A**, **B**, **C** refer to Table 1

**Fig. 9:**
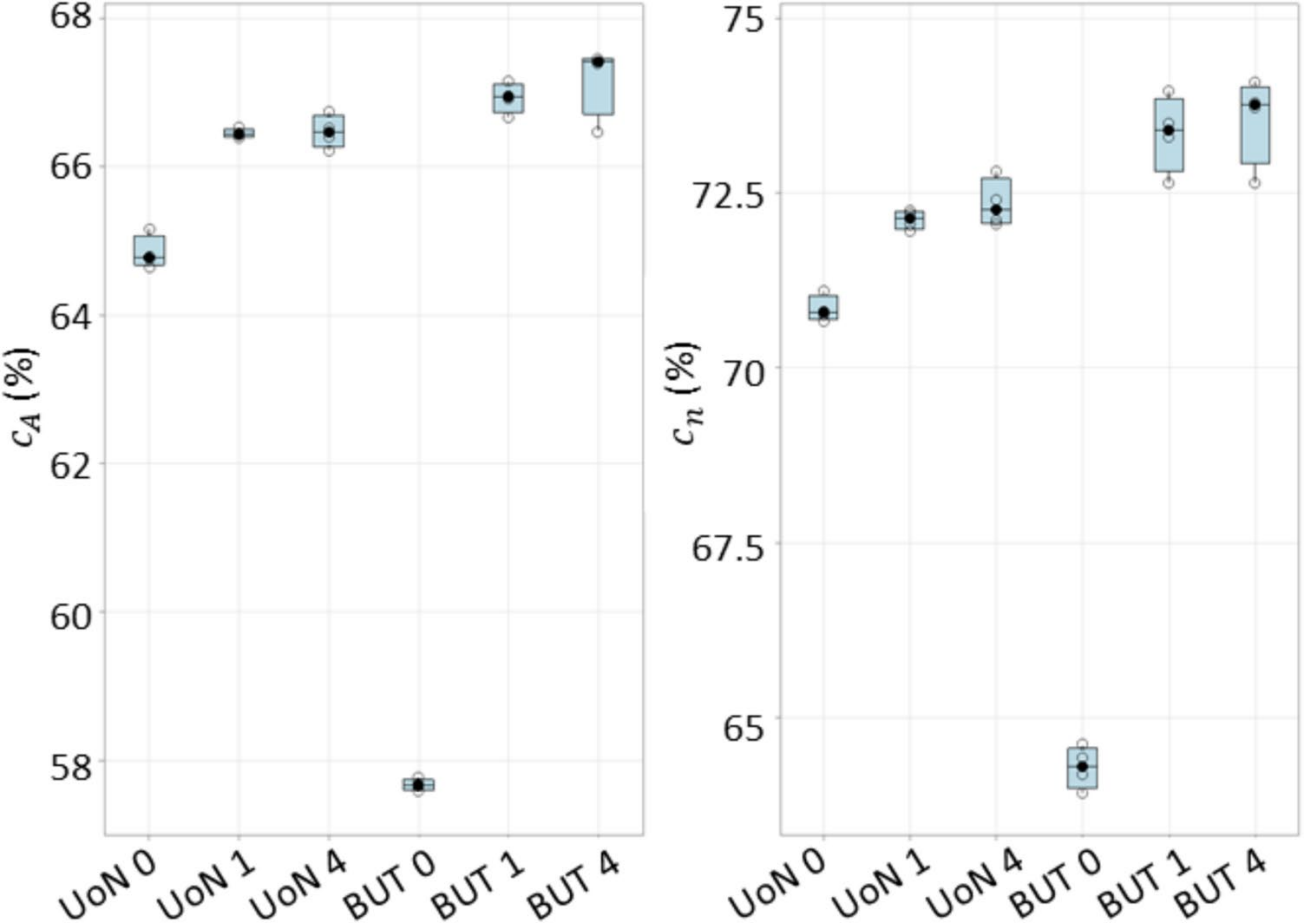
Boxplots showing part coverage; left: ${c}_{A}$ ratio of total area of sufficiently covered triangles over total area of all triangles; right: ${c}_{n}$ percentage of sufficiently covered triangles over total number of triangles. Horizontal axis: place of experiment (UoN: University of Nottingham; BUT: Brno University of Technology) and type of experiment: 0: no coating; 1: single coating before the four-repeats test; 4: coating layer re-applied at the beginning of each repeat

The part coverage results are summarised in Fig. 8, where a threshold was set to 75% of the maximum detected sampling density (see Sect. 2 and [26]) to visually discriminate between sufficiently covered and insufficiently covered triangles. Note that the outcome per triangle was averaged over four sampling density values obtained from the respective four measurement repeats. The boxplots of the part coverage indicator are reported in Fig. 9, expressed both as total area of sufficiently covered triangles over the total area of all triangles, and as percentage of sufficiently covered triangles over total number of triangles. Each point (observation) in the boxplots is representative of the result obtained from a single measurement; thus, temporal spacing and coated–uncoated conditions depend on the type of experiment (considering the three previously defined scenarios: (i) measurement repeats on uncoated surface; (ii) measurement repeats after single coating pass; (iii) coating repeats + measurement repeats, see Sect. 2).

**Fig. 10:**
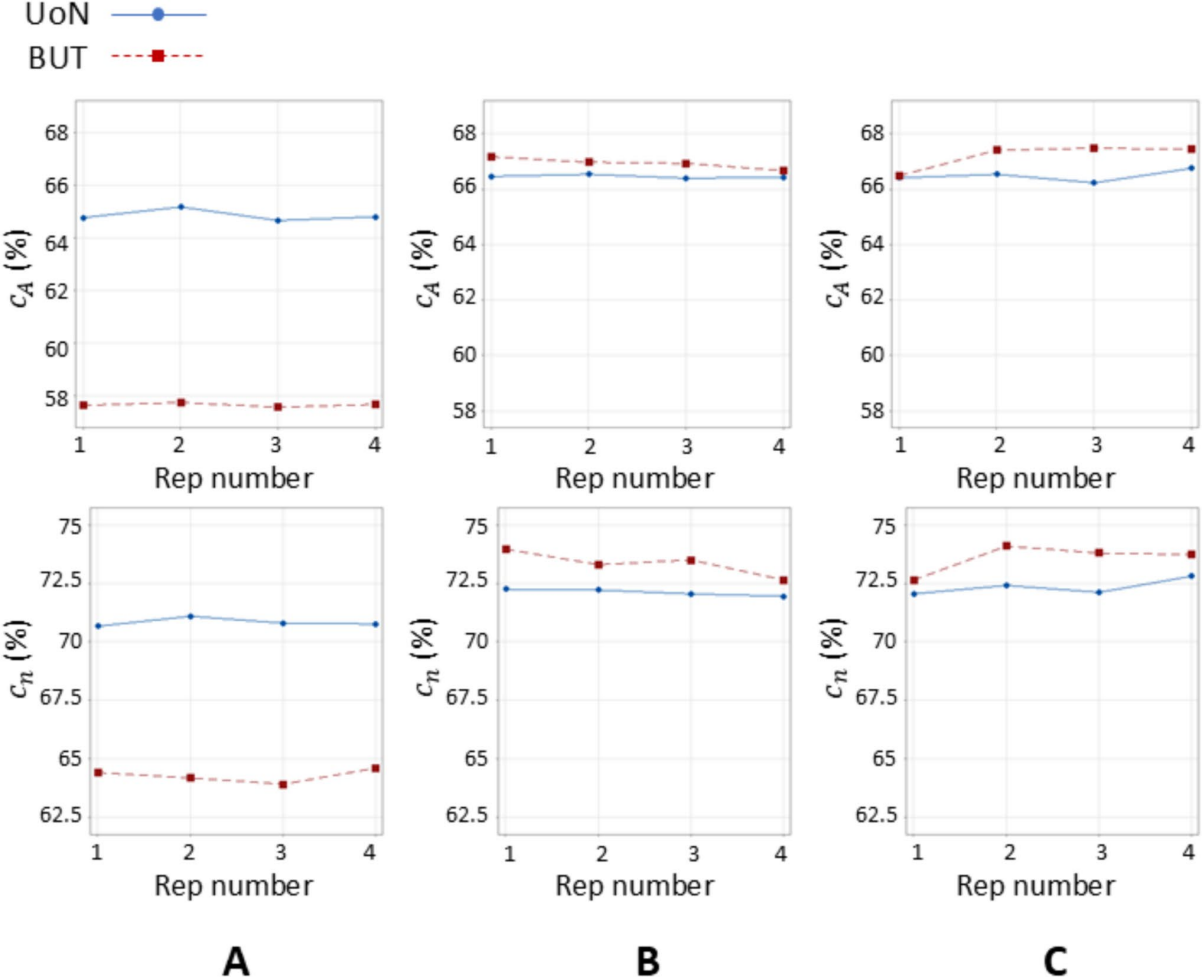
Time series plots of the part coverage indicator expressed as covered area ratio ${c}_{A}$ (top row) and percentage of covered triangles ${c}_{n}$ (bottom row). Strategies **A**, **B**, **C** refer to Table 1

Overall, Figs. 7 and 8 clearly show that the presence of coating is beneficial to both local sampling density and part coverage. In addition, repeated coating improves measurement performance compared to single coating, as the former approach (repeating the coating process) contrasts with the performance decay due to coating sublimation. Further information can be inferred by looking at the temporal behaviour of the part coverage indicator as highlighted in Fig. 10.

Finally, for the part coverage indicator, a one-way analysis of variance (ANOVA) for equal means resulted in the null hypothesis being rejected at the 0.05 significance level, with *p*-values equal to 1.07 × 10^−20^ and 1.10 × 10^−16^ for covered area and covered triangles respectively. This indicates that the presence-absence of coating, as well as whether the coating has just been applied or it has been applied since some time, are all relevant factors in affecting measurement performance in terms of local sampling density and part coverage.

**Fig. 11:**
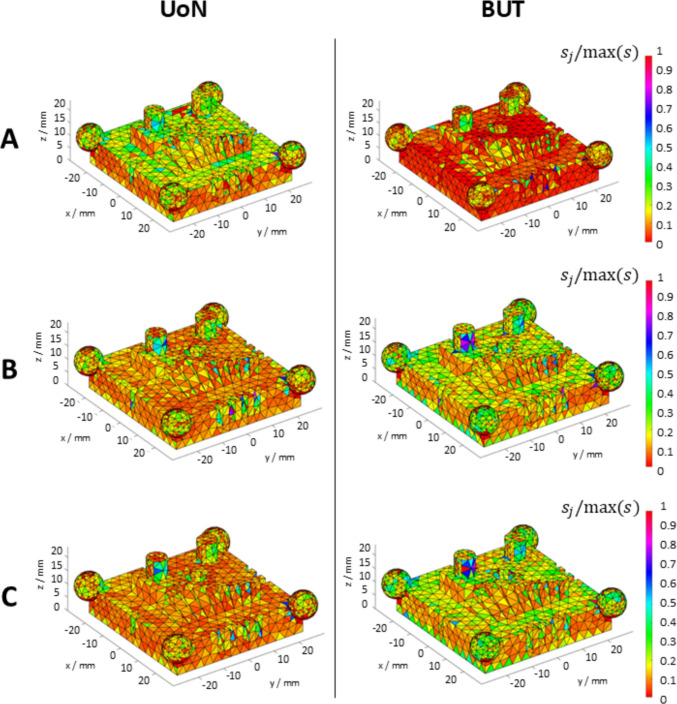
Example maps of triangles coloured on local sample dispersion (indicated with ${s}_{j}$). All local point dispersion values averaged over four repeats for UoN and BUT respectively. Strategies **A**, **B**, **C** refer to Table 1

**Fig. 12:**
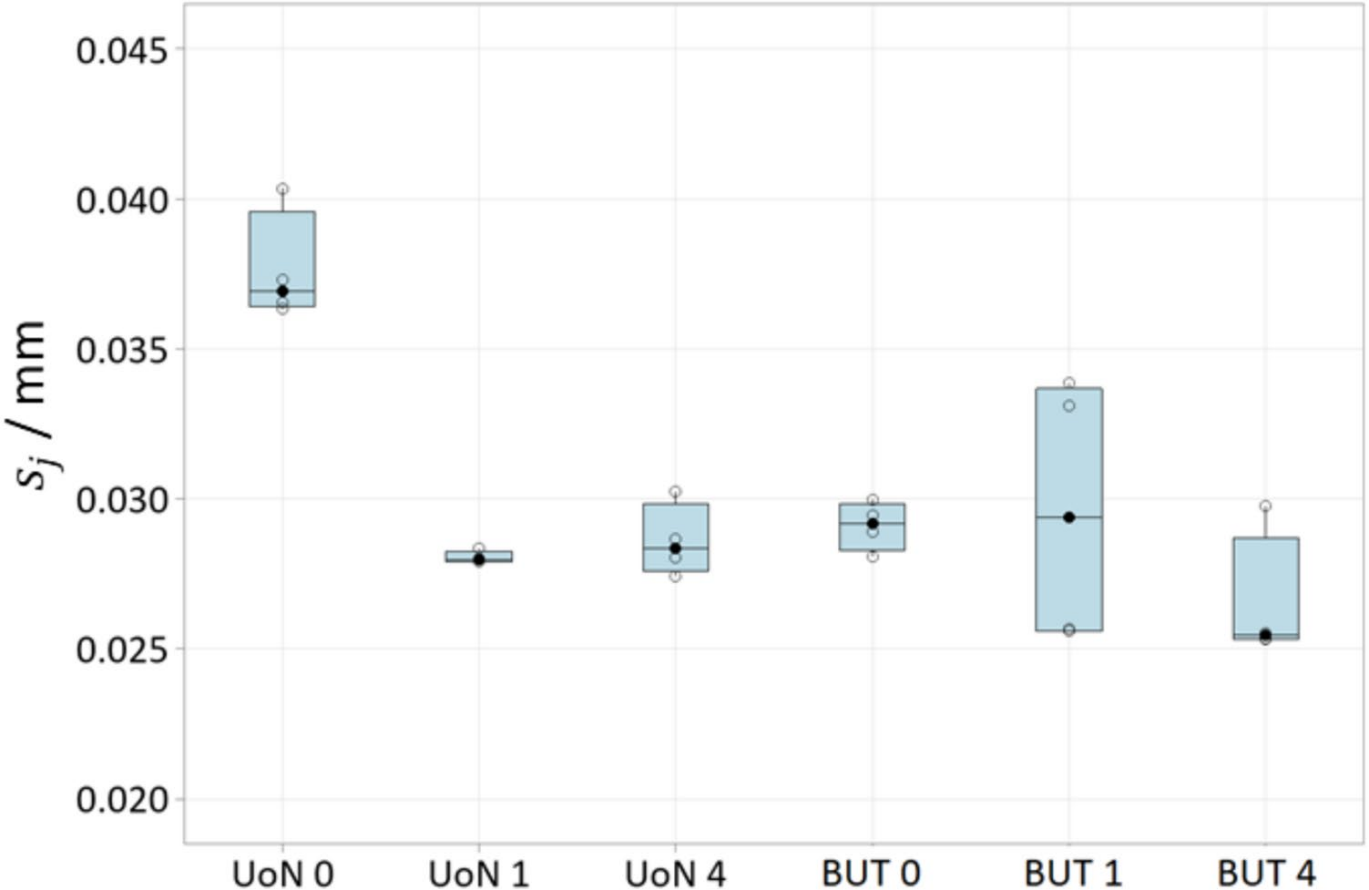
Boxplots of local sample dispersion (${s}_{j}$). UoN: University of Nottingham; BUT: Brno University of Technology; 0: no coating; 1: single coating before the four-repeats test; 4: coating layer re-applied at the beginning of each repeat

**Fig. 13:**
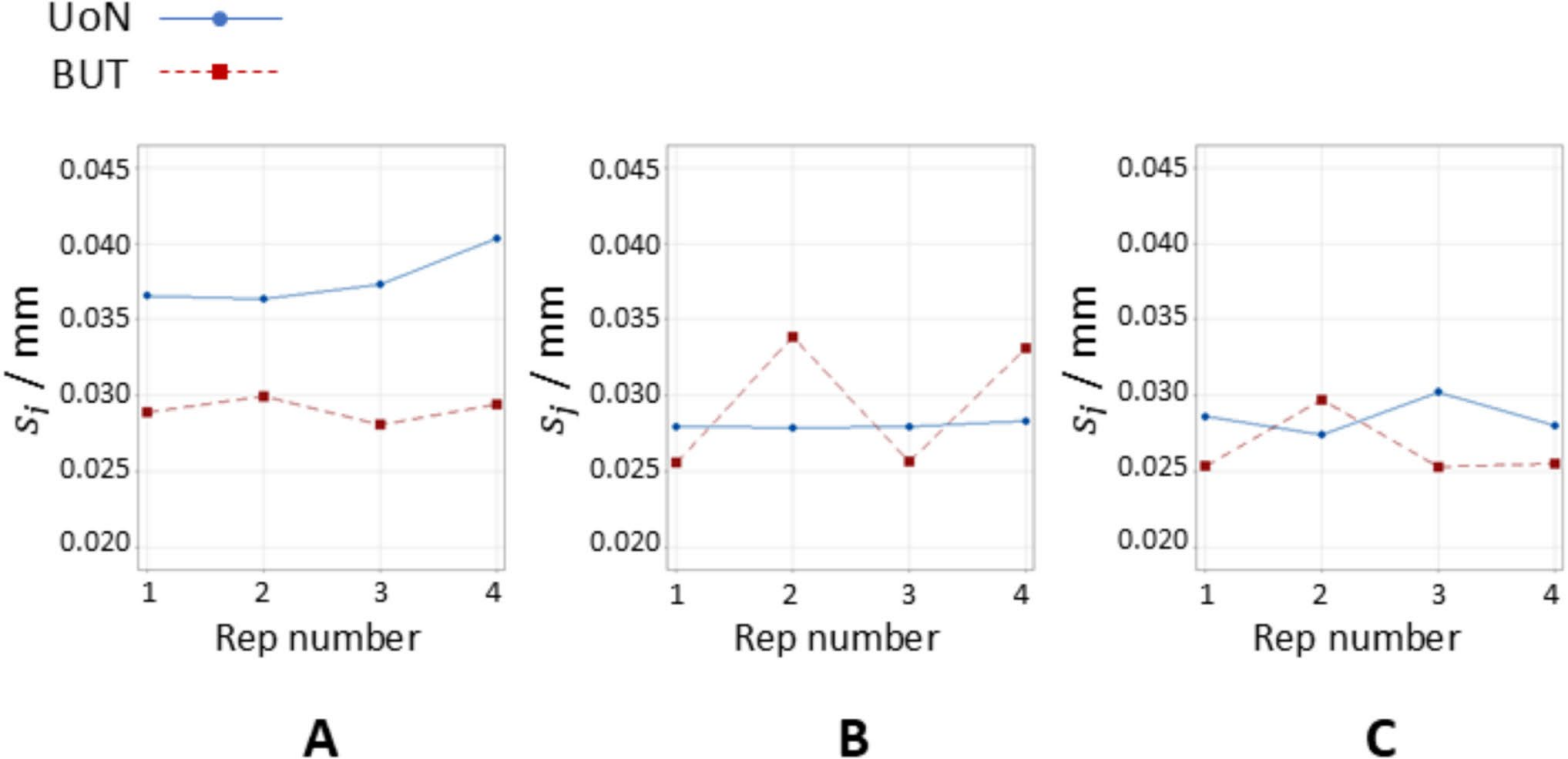
Time series plots of local sample dispersion (${s}_{j}$). UoN is indicated in blue and BUT in red. Strategies **A**, **B**, **C** refer to Table 1

The example colourmaps of point dispersion (or scatter) with respect to registered triangle mesh are shown in Fig. 11. Values for this indicator were normalised by division with the maximum point dispersion value recorded across all datasets. Figures 12 and 13 illustrate the boxplots and the time series plots of average local scatter, respectively, showing how the use of coating spray contributes in most cases to reduce the dispersion of the measured points compared to the uncoated measurements.

For the results shown in Fig. 12, ANOVA for equal means resulted in the null hypothesis being rejected at the 0.05 significance level, with *p*-value equal to 3.24 × 10^−5^.

### Local dispersion in the Gaussian field

**Fig. 14:**
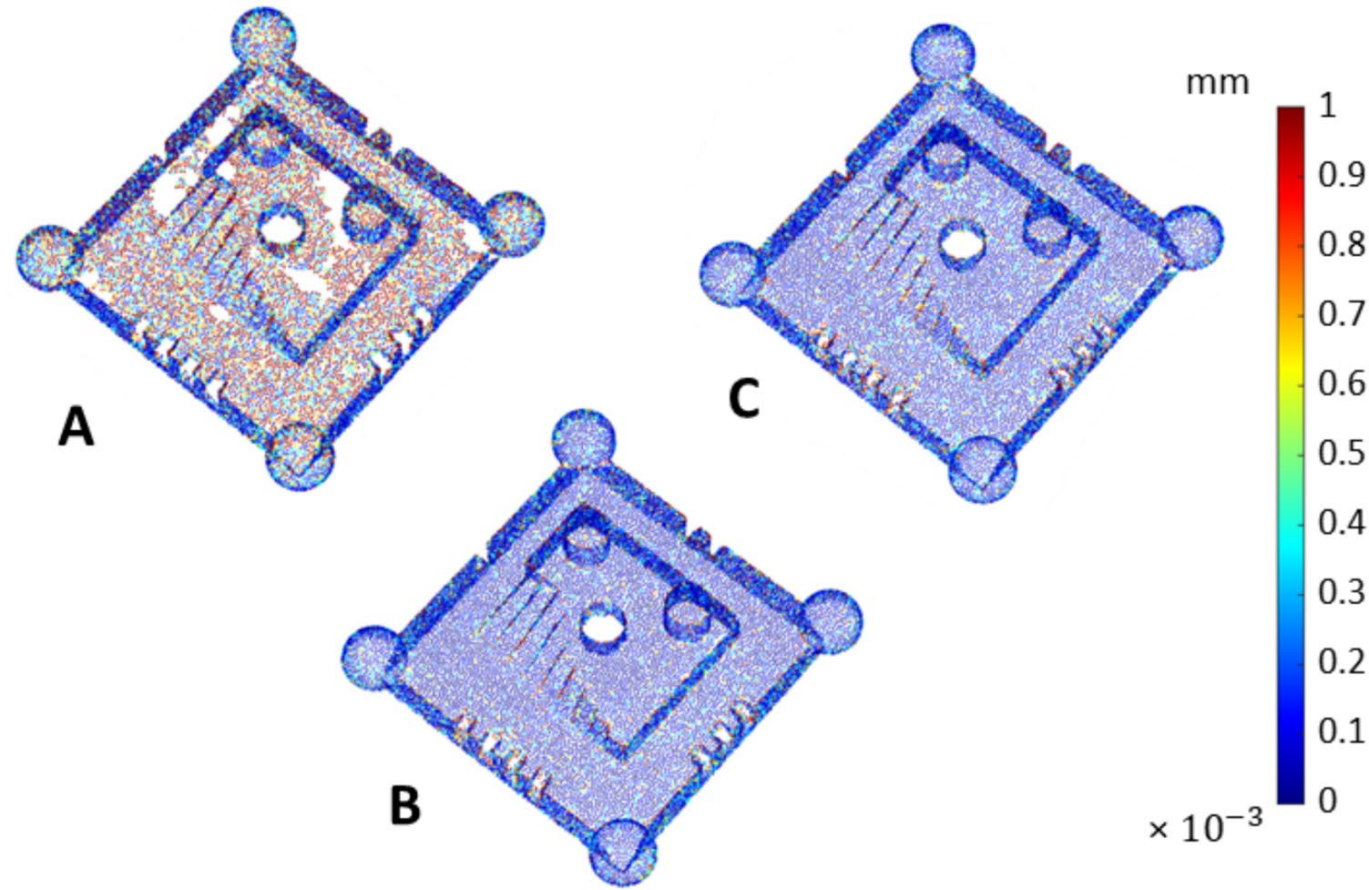
UoN campaign: spatial maps of local dispersion in the Gaussian field. The mean point cloud extracted from the Gaussian field of the UoN measurements is coloured according to local variance ${{\sigma }^{2}}_{GF}$. Strategies **A**, **B**, **C** refer to Table 1

**Fig. 15:**
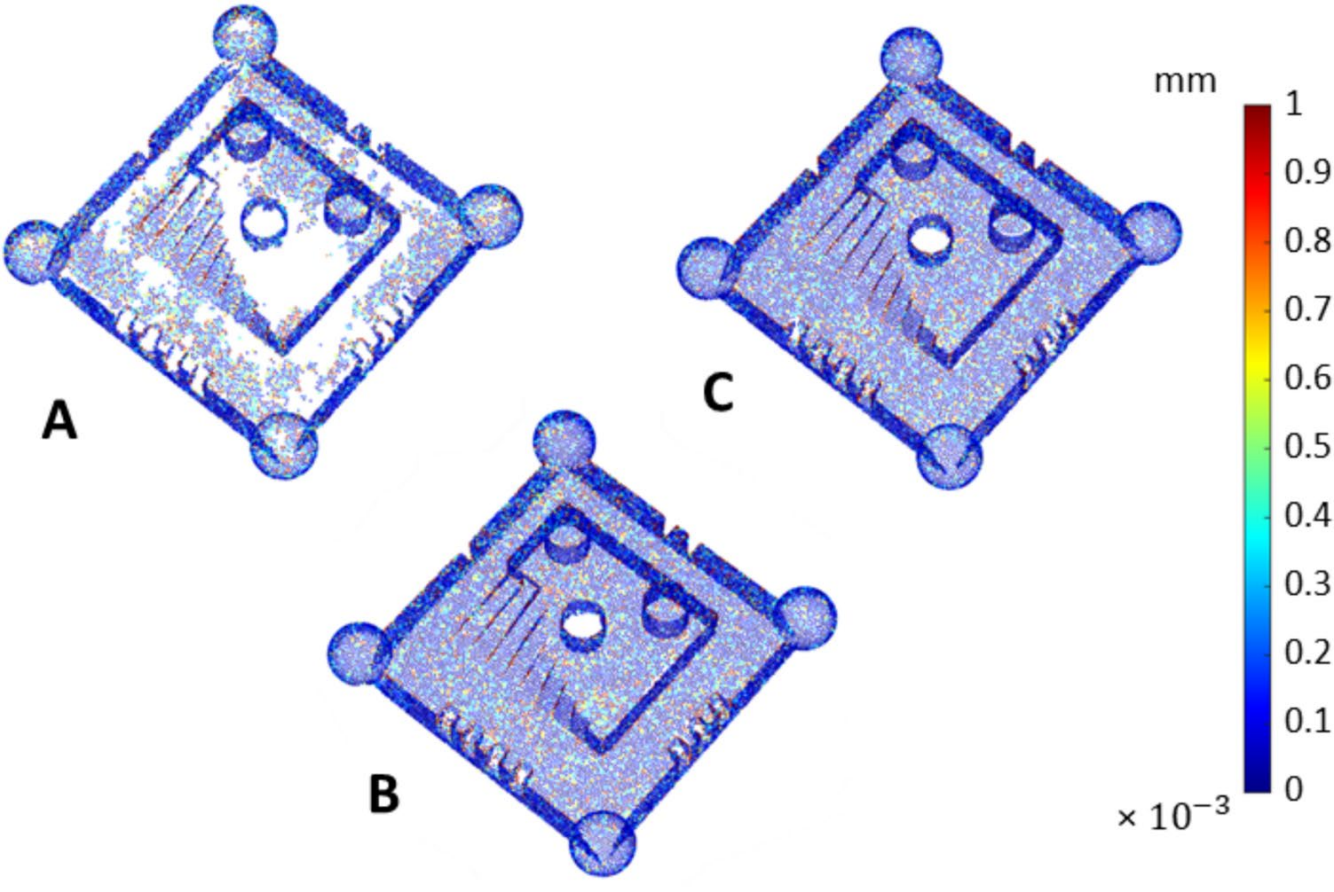
BUT campaign: spatial maps of local dispersion in the Gaussian field. The mean point cloud extracted from the Gaussian field of the BUT measurements is coloured according to local variance ${{\sigma }^{2}}_{GF}$. Strategies **A**, **B**, **C** refer to Table 1

**Fig. 16:**
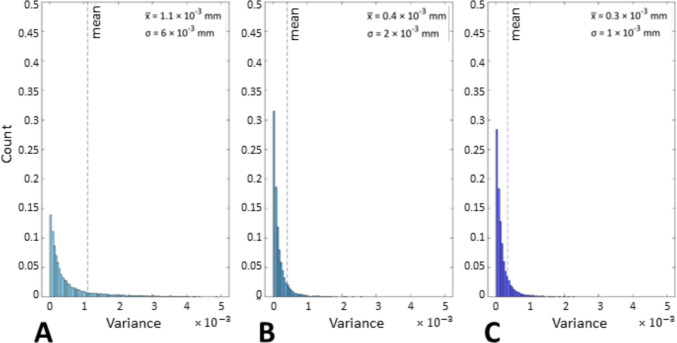
UoN campaign: distributions of local dispersion in the Gaussian field with mean $\overline{x }$ and standard deviation σ. Strategies **A**, **B**, **C** refer to Table 1

**Fig. 17:**
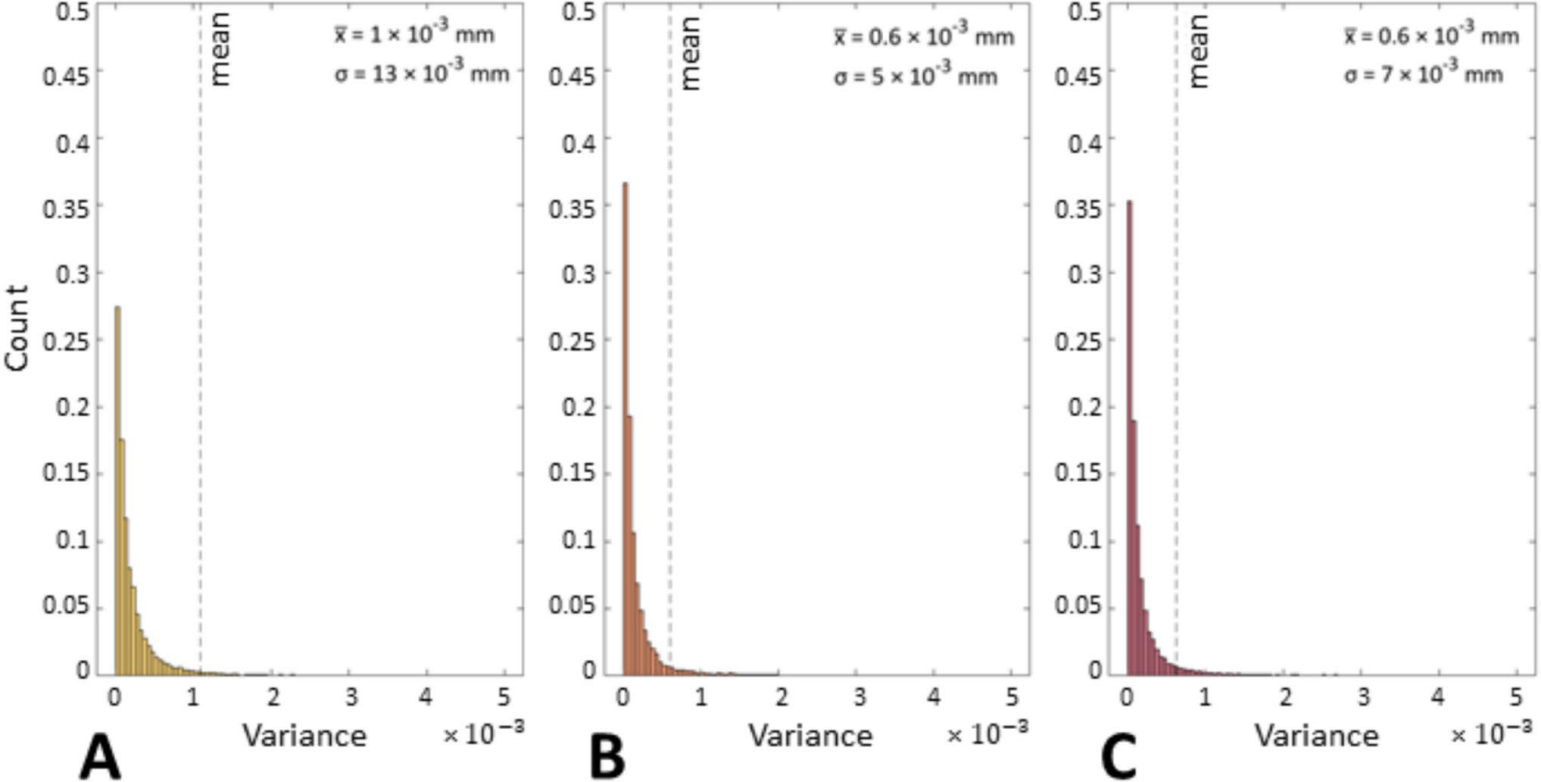
BUT campaign: distributions of local dispersion in the Gaussian field with mean $\overline{x }$ and standard deviation σ. Strategies **A**, **B**, **C** refer to Table 1

In addition to the indicators shown in Sect. 3.1, the method developed in [27] was applied to prove that the application of coating spray increases the precision of the measurements. Figures 14 and 15 show the spatial maps of the local dispersion in the Gaussian field (local variance σ) extracted from the statistical model of the UoN measurements and BUT, respectively, for each coating application strategy, whereas Figs. 16 and 17 show the related distributions.

### Dimensional analysis for selected geometric features

**Fig. 18:**
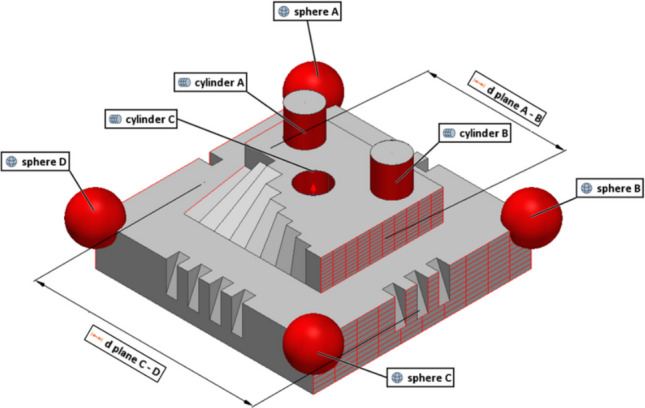
Digital 3D model showing the selected features

In order to test how the use of coating affects the precision in dimensional measurements, selected features were extracted from datum geometries locally fitted to the point clouds using least-squares (as summarised in Fig. 18).

**Fig. 19:**
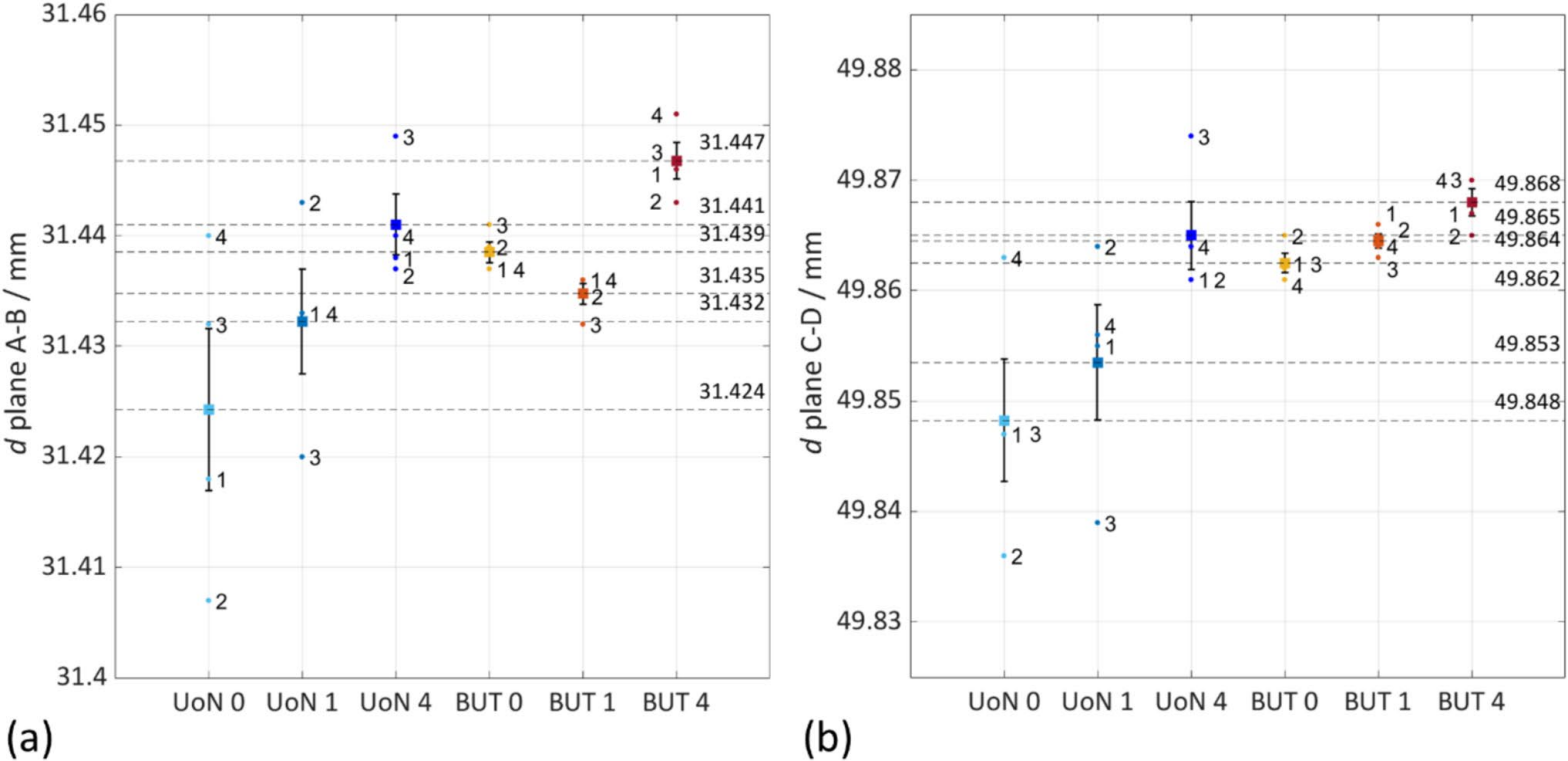
Selected features interval plots: standard uncertainties for each coating application, as reported in Table 7. (**a**) Distance plane A-B. (**b**) Distance plane C-D

**Fig. 20:**
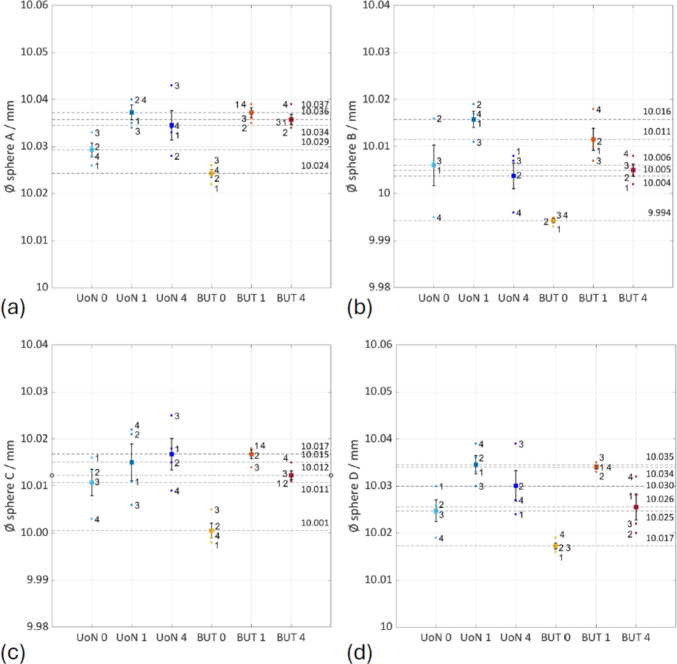
Selected features interval plots: standard uncertainties for each coating application, as reported in Table 7. (**a**) Diameter sphere A. (**b**) Diameter sphere B. (**c**) Diameter sphere C. (**d**) Diameter sphere D

**Fig. 21:**
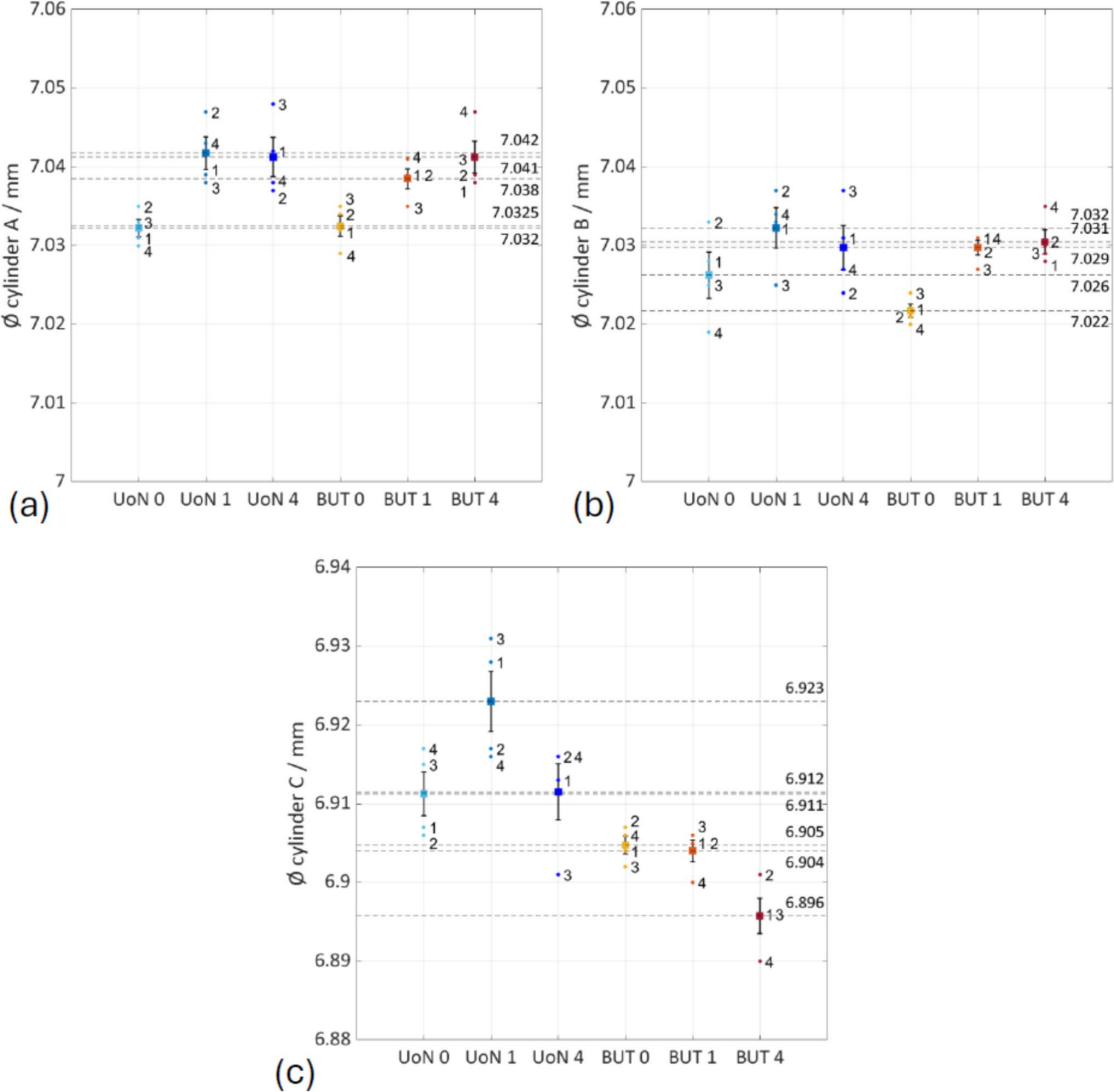
Selected features interval plots: standard uncertainties for each coating application, as reported in Table 7. (**a**) Diameter cylinder A. (**b**) Diameter cylinder B. (**c**) Diameter cylinder C

Based on the Good Practice Guide No. 130 [38], the standard uncertainty (i.e., the standard error of the mean computed on dimensions extracted by fitting point clouds to appropriate datums) in the selected dimensions was calculated as discussed in [26] and indicated as ${u}_{\mathrm{M}}$

5 $${u}_{\mathrm{M}}= {S}_{{\mu }_{x}}=\frac{s}{\sqrt{n}}$$

where ${S}_{{\mu }_{x}}$ is the standard error of the mean, $s$ is the sample standard deviation computed on the $n$ repeats, $\overline{x }$ is the mean of the $n$ results for the dimensional features and $M$ is the measurement solution (0: no coating; 1: single coating before the four-repeats test; 4: coating layer re-applied at the beginning of each repeat). The results are shown in Figs. 19, 20, and 21 and summarised in Table 7.

**Table 7:** Results for the features/mm

Feature	Nominal	$\overline{x}\pm {u }_{\mathrm{UoN}0}$	$\overline{x}\pm {u }_{\mathrm{UoN}1}$	$\overline{x}\pm {u }_{\mathrm{UoN}4}$	$\overline{x}\pm {u }_{\mathrm{BUT}0}$	$\overline{x}\pm {u }_{\mathrm{BUT}1}$	$\overline{x}\pm {u }_{\mathrm{BUT}4}$
*d* plane A-B	31.500	31.424 ± 0.007	31.432 ± 0.005	31.441 ± 0.003	31.439 ± 0.001	31.435 ± 0.001	31.447 ± 0.002
*d* plane C-D	50.000	49.848 ± 0.005	49.853 ± 0.005	49.865 ± 0.003	49.862 ± 0.001	49.864 ± 0.0006	49.868 ± 0.001
Ø sphere A	10.000	10.029 ± 0.002	10.037 ± 0.002	10.034 ± 0.003	10.024 ± 0.001	10.037 ± 0.001	10.036 ± 0.001
Ø sphere B	10.000	10.006 ± 0.004	10.016 ± 0.002	10.004 ± 0.003	9.994 ± 0.0005	10.011 ± 0.002	10.005 ± 0.001
Ø sphere C	10.000	10.011 ± 0.003	10.015 ± 0.004	10.017 ± 0.003	10.001 ± 0.0015	10.017 ± 0.001	10.012 ± 0.001
Ø sphere D	10.000	10.025 ± 0.002	10.035 ± 0.002	10.030 ± 0.003	10.017 ± 0.001	10.034 ± 0.001	10.026 ± 0.003
Ø cylinder A	7.000	7.032 ± 0.001	7.042 ± 0.002	7.041 ± 0.002	7.033 ± 0.001	7.038 ± 0.001	7.041 ± 0.002
Ø cylinder B	7.000	7.026 ± 0.003	7.032 ± 0.003	7.029 ± 0.003	7.022 ± 0.001	7.029 ± 0.001	7.031 ± 0.002
Ø cylinder C	7.000	6.911 ± 0.003	6.923 ± 0.004	6.912 ± 0.004	6.905 ± 0.001	6.904 ± 0.001	6.896 ± 0.002

Separate one-way ANOVA tests were run for each feature to check for equal means at *α* = 0.05. In every case, the null hypothesis was rejected, as reported in Table 8.

**Table 8:** Results for p-values

Distances (Fig. 19)	Spheres (Fig. 20)	Cylinders (Fig. 21)
d_plane A–B: p = 1.17 × 10⁻2 d_plane C–D: p = 4.70 × 10⁻3	Ø A: p = 1.61 × 10⁻4 Ø B: p = 2.06 × 10⁻4 Ø C: p = 2.10 × 10⁻3 Ø D: p = 1.65 × 10⁻4	Ø A: p = 1.60 × 10⁻3 Ø B: p = 3.13 × 10⁻2 Ø C: p = 3.62 × 10⁻5

### Coating thickness analysis on the Si-wafer

**Fig. 22:**
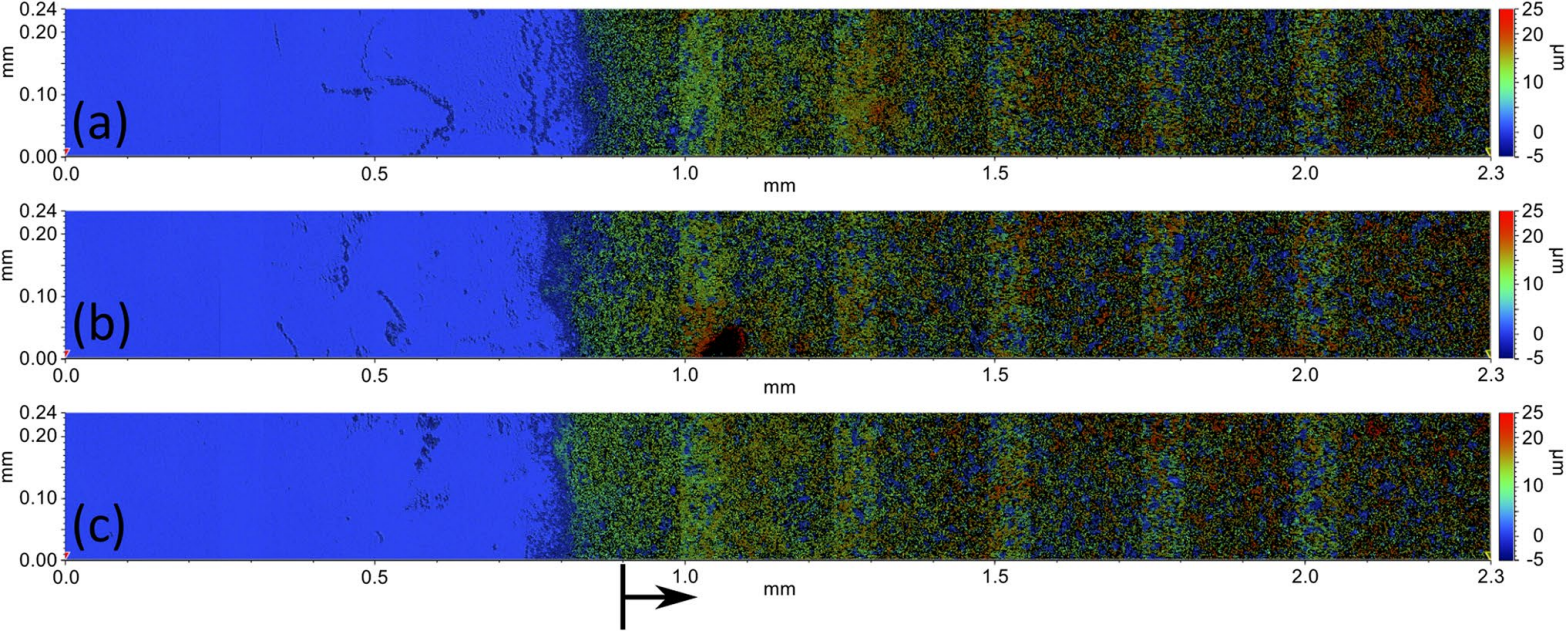
Surface texture measurement of the Si-wafer with indication of the area used for the coating thickness evaluation. (**a**) Location 1. (**b**) Location 2. (**c**) Location 3

To check whether the application of the coating layer influences the variation in the measurement of the selected linear dimensions (for instance the diameter of the spheres), the thickness of the matting material was measured using a 3D optical profilometer as explained in Sect. 2.5. The measured data acquired from three locations on the Si-wafer is shown in Fig. 22, where an area of (0.24 × 1.40) mm was selected for thickness evaluation (indicated by the black arrow in the figure). The periodical vertical bands in the figure result from the stitching of individual scans (see Fig. 6 for a schematic representation).

**Fig. 23:**
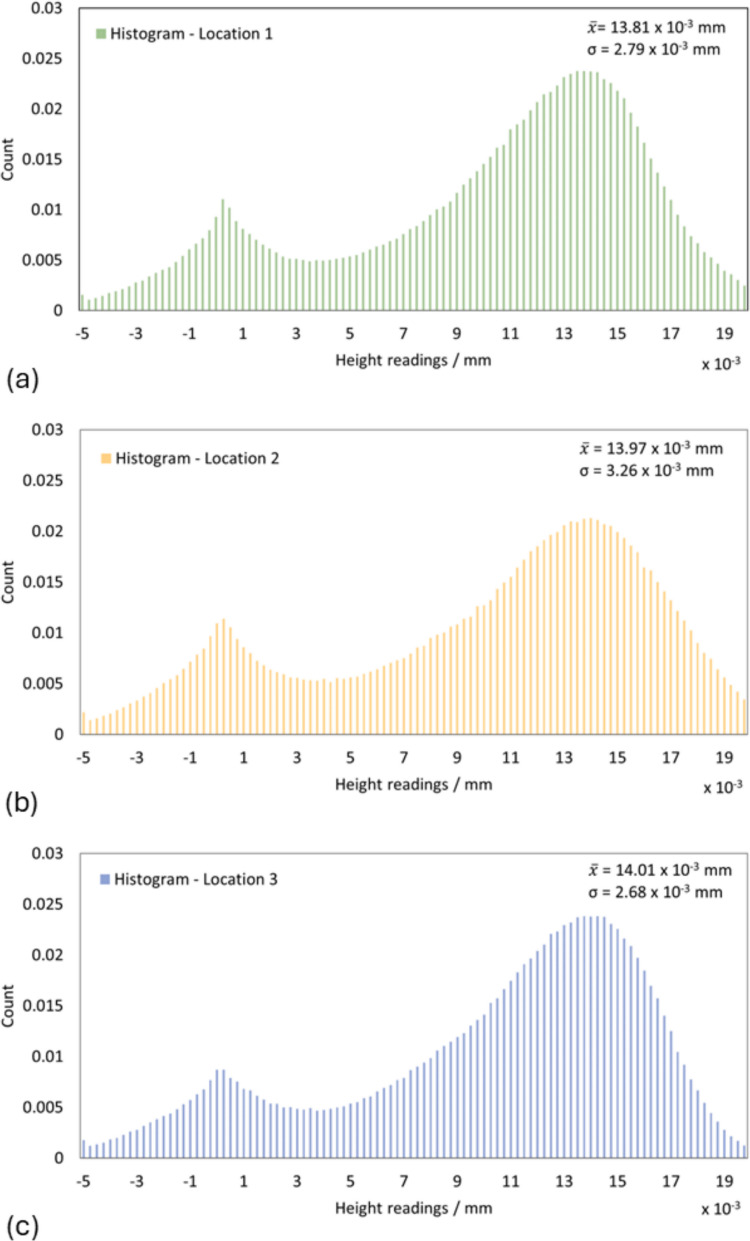
Histograms of height readings at three selected locations on the Si-wafer, as indicated in Fig. 22. (**a**) Location 1. (**b**) Location 2. (**c**) Location 3

Histograms of the measured heights were obtained for each selected location, as shown in Fig. 22. The matte coating did not cover the surface in its entirety and, therefore, the measured data contained a significant number of null values. For this reason, only the data of the abovementioned selected interval was fitted. The histograms of the height for the three regions showed a good agreement, and an average value of (13.92 ± 2.88) µm was obtained for the deposited layer thickness (Fig. 23). The average value was chosen to evaluate the thickness of the matting spray based on experience and a similar approach from our previous study [15].

### Surface texture analysis

**Fig. 24:**
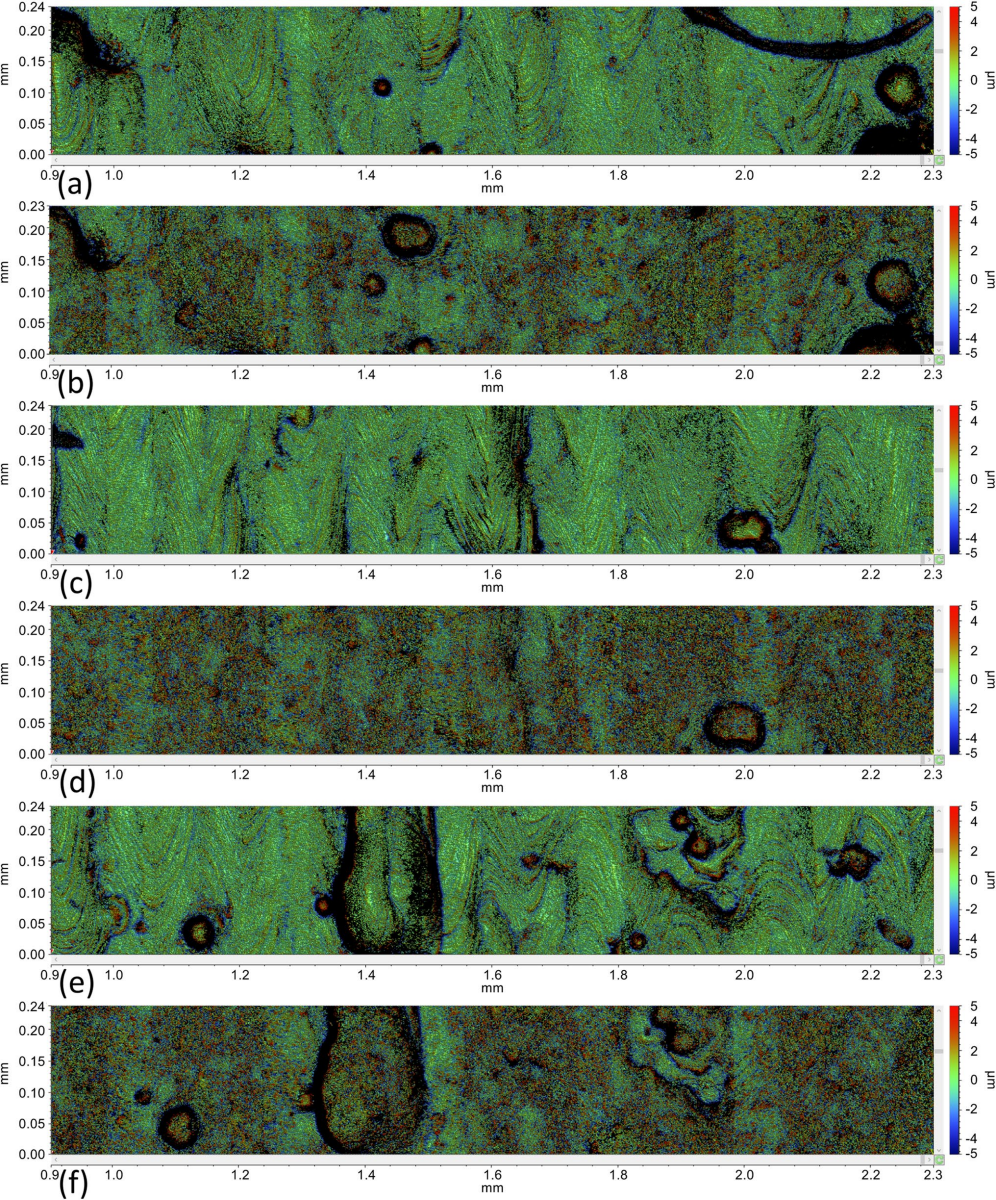
Surface texture measurement at three selected locations, uncoated and coated. Location 1 — (**a**) uncoated and (**b**) coated; location 2 — (**c**) uncoated and (**d**) coated; location 3 — (**e**) uncoated and (**f**) coated

In order to assess whether the application of the matting spray caused a change in the characteristics of the surface texture of the measured part, measurements of selected regions of interest were obtained pre and post application of the coating, more specifically in correspondence of the upper surface of one of the cylindrical features previously indicated in Fig. 5b. The results of the measurements after zero mean (F-operation) and Gaussian Regression Filtering with Short Wavelength Pass (S-Filter = 0.025 mm) pre and post spray application in correspondence of three selected locations are shown in Fig. 24.

The macroscopic formations visible in Fig. 24 are the result of PBF-LB processing of the test artefact surface and were removed by Gaussian Regression Filter described earlier ahead of computing areal texture parameters following ISO 25178–2 [39]. The material ratio parameters (Spk, Svk and Sk) and ratios between them describe the peak, valley and core material of a surface. They are used as a good indication of changes in the surface texture caused by the coating when comparing uncoated and coated surfaces. The values are summarised in Table 9.

**Table 9:** Functional surface texture parameters for three selected locations. *Sk* — core height; *Spk* — reduced peak height; *Svk* — reduced valley depth; *Spk/Sk* — ratio of the reduced peak height to core roughness; *Svk/Sk* — reduced valley depth to core roughness; *Smr(c)* — areal material ratio; *SMr1* — peak material portion; *SMr2* — valley material portion

	Location 1 uncoated	Location 1 coated	Location 2 uncoated	Location 2 coated	Location 3 uncoated	Location 3 coated
Sk/mm	3.07 × 10^−3^	5.99 × 10^−3^	2.66 × 10^−3^	6.80 × 10^−3^	2.93 × 10^−3^	6.75 × 10^−3^
Spk/mm	5.01 × 10^−3^	4.97 × 10^−3^	2.65 × 10^−3^	2.50 × 10^−3^	3.08 × 10^−3^	3.29 × 10^−3^
Svk/mm	5.11 × 10^−3^	6.05 × 10^−3^	2.73 × 10^−3^	4.20 × 10^−3^	3.54 × 10^−3^	4.82 × 10^−3^
Spk/Sk/-	1.631	0.829	0.994	0.367	1.053	0.488
Svk/Sk/-	1.664	1.009	1.024	0.618	1.209	0.715
Smr(c)/%	49.85	53.55	50.55	53.75	51.45	53.35
SMr1/%	12.06	8.68	11.75	7.011	12.51	8.13
SMr2/%	87.52	87.10	87.90	87.76	86.98	87.58

## Discussion

The proposed work focused on exploring how the application of a sublimating matte spray can improve the performance of optical measurement of metal additively manufactured parts. A series of measurement campaigns were performed using two fringe projection systems, and the results were recorded pre and post application of the coating layer. Quality of measurements was assessed using custom indicators (i.e., rate of surface coverage, level of sampling density and local point dispersion).

Local sampling density and part coverage together describe to what extent the optical system is able to capture the surface in terms of completeness and uniformity with or without coating. Part coverage represents the proportion of the nominal surface whose mesh facets satisfy a predefined point-density threshold. In the uncoated part, reflections reduce the point count on many facets and lower overall coverage. Once the sublimating matte layer is applied, the reflections are suppressed, nearly all facets meet the threshold, demonstrating that the coating improves surface visibility. Local sampling density complements the coverage indicator by capturing how uniformly points are distributed over the surface. For each mesh facet, the number of points per square millimetre is computed; this value highlights whether difficult-to-access regions (for example deep recesses, sharp edges) show the same sampling as easily accessible facets. From the obtained results, it was possible to observe that the use of the coating generally increases the sampling density and rate of coverage as expected, and as clearly visible from the obtained results (colormaps in Figs. 7 and 8). Density maps show that, after coating, even those previously under-sampled areas approach densities close to the overall mean. This result indicates that the coating improves sampling uniformly across the entire geometry rather than only in selected zones. Regions of low density are consistently located on steep vertical walls, in recessed pockets, and along inner corners where the systems’ line-of-sight could have been potentially and partially occluded. In particular, the uncoated BUT data in Fig. 8 shows a large zone on the central top surface where no points were captured, likely caused by strong reflections that blinded the sensor and blocked data acquisition. The same area becomes uniformly sampled once the sublimating spray is used (with both strategies B and C). After the application of the spray coating, an improvement in point density and coverage can be observed on surfaces prone to glare, whereas difficult-to-access features such as deep pockets remain challenging for both systems. As apparent from the boxplots of the covered area and triangles (Fig. 9), the use of a single coating application ahead of executing four measurement repeats appeared to generate less dispersed results for both measuring instruments at UoN and BUT, compared to the tests carried out with the coating layer re-applied at the beginning of each measurement repeat, the BUT results appearing more dispersed compared to the UoN ones. Although with a higher dispersion, the re-application of the coating per repeat showed the highest percentages of coverage, especially in the BUT campaigns. The information for the rate of coverage reported above was confirmed by the time series plot (Fig. 10), where the contribution over time of the sublimating matte spray at each measurement repeat is shown. The decrease in the rate of coverage over time for a single coating application (especially for the BUT tests) confirmed the sublimating nature of the spray. The point dispersion, locally assigned to each triangle in the reference 3D model, appeared to slightly improve (i.e., decrease) with the use of the coating (Fig. 11), especially for the UoN tests. The dispersion maps support the density results observed in Fig. 7: zones of poor coverage generally coincide with elevated dispersion, showing that few measured points also affect positional repeatability. It is worth pointing out that in correspondence of some filleted edges high dispersion is noticeable even when density is satisfactory; this underlines that geometric discontinuities can affect precision independently of coverage. From the boxplots of local dispersion (Fig. 12), the best result was found to be associated with the use of a single layer of coating at the beginning of the entire test (for four measurement repeats). The tests carried out at BUT showed less precise results, but the use of coating spray seemed to generally reduce the average local scatter.

The local point dispersion indicator captures the variability of measured points. Low dispersion means that, across repeats, points are positioned consistently at the same depth; high dispersion flags unstable regions where optical artefacts cause positional scatter. The coating is effective when dispersion decreases across the entire surface and the high-variance regions that previously indicated specular spots disappear. In particular, the distributions of point variance (Figs. 14 and 15) showed more precise results as layers of coating were added. A net difference between the uncoated measurements and the coated ones is visible, as precision is much higher in the latter cases for both campaigns (i.e., standard deviation appears to decrease significantly when the spray coating is applied). The most precise result is represented by the measurements obtained when the coating layer was re-applied at the beginning of each repeat for the UoN tests (Fig. 14). In the BUT cases shown in Fig. 15, the best result was given by the application of a single coating layer at the beginning of the entire test (for four measurement repeats). The statistical data and distributions shown in Figs. 16 and 17 confirm the slight but genuine differences between the maps in Figs. 14 and 15, especially for strategy A, which shows a lower concentration of high-variance zones in the BUT dataset.

Selected linear features were observed to evaluate how the use of coating layer sprayed onto the surface of an object might influence the measurement of linear dimensions, especially for a complex shape with non-smooth surface such as an AM-built sample. For instance, the average differences of 0.0039 mm and 0.0079 mm between the radius of the uncoated and the coated spheres could be observed in the UoN and BUT measurement campaigns respectively (more specifically in the case of a single coating application throughout the measurement repeats). These results led to approximately half the measured thickness of the matte coating spray obtained from the tests carried out with the Si-wafer, which was found to be approximately 13.92 µm. As can be interpreted from Figs. 19, 20, and 21 and results in Table 7, there is an average shift across the different coating application strategies that drops from approx. 8 µm (0 — no coating) to 5 µm (1 — single coating) and 4 µm (4 — re-coating), implying that the matte layer suppresses reflectance-induced errors. Across the nine features selected, the mean signed UoN–BUT difference is negligible (− 1 to − 2 µm) under all coating strategies, indicating no systematic scale offset. On the other hand, averaged across all strategies, inter-sensor bias varies by feature type: plane-to-plane distances measured with the BUT system are approx. 9 µm larger than those from the UoN scanner, 4 µm for spheres, and 6 µm for cylinders. This indicates a systematic overestimation by BUT on extended planar features and underestimation on smaller curved geometries.

Examining the datasets by strategy, the following observations emerge:

1. No coating shows the greatest dispersion in all linear dimensions, due to optical artefacts during measurement.
2. Single coating produces the tightest distributions. This confirms that a single, thin matte layer is sufficient to stabilise point acquisition over at least four measurement repeats.
3. Re-coating at each repeat introduces bias and some extra scatter because each freshly added layer of coating builds on the surface. The growing positive bias is accompanied by a moderate increase in variance, indicating that spray thickness is likely not perfectly uniform between applications.

In summary, the issue with the thickness could be caused not only by the clear presence of measurement error but also by error generated by the uneven application of the spray on the different surfaces of the part. Even with an attempt for uniform application, it is inevitable that some surfaces (typically the top surfaces) will present a thicker layer of the spray material compared to other areas of the object (for instance the side surfaces).

Referencing each mean measurement to the CAD nominal shows that the uncoated measurements remain within 9 µm deviation, whereas a single matte layer produces a systematic shift of approx. 4 µm, compatible with about 2 µm of material on each side of the feature; repeated coating applications push this deviation to approx. 14 µm, highlighting the potential accumulation of spray layers and demonstrating that comparisons with the nominal geometry can isolate coating build-up from instrument error.

Further tests on surface texture were performed using an optical profilometer to evaluate the thickness of the coating layer and how the characteristics of the surface might be affected by the presence of the matting spray. For the coating thickness analysis, the distribution of the coating thickness measured at three selected locations on the smooth planar surface of the Si-wafer was relatively wide, meaning that the matt coating had likely been unevenly sprayed over the surface of the object, even though the application was performed under controlled conditions. For the AM artefact, an attempt was made to maintain the conditions of the coating application as uniform as possible onto the entirety of the surface. As previously mentioned, the complexity of the object shape and the nature of the spray application might result in layer thicknesses likely to vary at different surface locations on the part.

For the analysis of surface texture characteristics, some observations can be made: *Sk* increased with the application of the coating, indicating that the matte spray increases the surface roughness; *Spk* appeared to be almost unchanged with the application of the coating, meaning that the average height of the peaks is maintained. On the other hand, the *Spk/Sk* ratio decreased with coating application, indicating that mainly the core roughness of the surface increased. An increase in the area with the peaks can be observed from the increment of the *SMr1* parameter; *Svk* slightly increased and may indicate an increase in surface roughness in the valley (i.e., the matting material is probably not filling the valleys). At the same time, the *Svk/Sk* ratio decreased, indicating again a growth of the core roughness. The *SMr2* parameter is almost constant, meaning unchanged areas in the valleys.

## Conclusions

The proposed research aimed at investigating the impact of applying a sublimating matting spray onto the surface of additively manufactured parts. The matte spray effectively reduces the reflections caused by the metal surfaces, making it easier to capture more complete measurements and improve the precision of optical measurements. In this work, the effectiveness of a sublimating matting spray in enhancing optical measurements was demonstrated by applying a series of performance indicators on point cloud data obtained via fringe projection. After application of the sublimating matte coating, the rate of surface coverage reached almost completeness, and the point-wise dispersion consistently decreased. Part coverage raised from approx. 85–92% (uncoated part) to 98–100% (coated part), and local sampling density appeared to be uniform even in correspondence with difficult-to-access regions/complex areas. The coating thickness was measured to be within a few micrometres without inducing any statistically significant dimensional bias, although an offset in the measurement of a radius revealed the effects of uneven spray applications. The surface texture analysis shows that the coating raises core roughness (higher *Sk*), leaves peak height virtually unchanged (*Spk*), and enlarges valley roughness only slightly (*Svk*), while the valley area itself remains essentially constant (*SMr2*). The result is a uniform matte finish that does not clog surface recesses. In general, it is worth noting that the effectiveness of the matting spray depends on factors such as the spray application technique chosen when carrying out the tests: reapplying the spray before each acquisition produced the narrowest variance distributions, whereas a single initial layer offered a good balance between precision and speed during several subsequent measurements.

In summary, key points are:

- Coverage and density: nearly the entirety of the visible area is captured after coating, eliminating data gaps caused by specular reflections; time-series plots confirm gradual loss of coverage for a single application owing to sublimation.
- Precision and variance: local standard deviation is reduced considerably across all tests, demonstrating high repeatability and reduction in outliers; the best results are obtained from a single initial layer (in both campaigns), while re-spraying maximises coverage but yields higher dispersion.
- Dimensional bias: the presence of coating does not introduce any statistically significant dimensional bias, as for example the radius shifts of approx. 4–8 µm remain within half the measured coating thickness (13.92 µm), indicating only a small, controllable bias from the coating layer.
- Surface-texture effects: coating raises core roughness yet leaves peak height (*Spk*) and valley area (*SMr2*) largely unchanged, confirming a uniform matte finish.

In conclusion, the application of a sublimating matting spray appears to significantly enhance measurement quality, ultimately leading to reduced rework and enhanced product performance. Future work should refine coating spray application strategies for uniform thickness control on complex shapes and extend validation to additional materials and optical systems. In addition, future research should systematically investigate how changes in mesh step size and other tessellation parameters affect each proposed indicator, to develop formal guidelines for selecting an optimal mesh resolution.
